# Integrated stress response signaling acts as a metabolic sensor in fat tissues to regulate oocyte maturation and ovulation

**DOI:** 10.1016/j.celrep.2024.113863

**Published:** 2024-03-07

**Authors:** Lydia Grmai, Manuel Michaca, Emily Lackner, Nampoothiri V.P. Narayanan, Deepika Vasudevan

**Affiliations:** 1 Department of Cell Biology, University of Pittsburgh School of Medicine, Pittsburgh, PA, USA; 2 Department of Psychiatry and Behavioral Sciences, Duke University Medical Center, Durham, NC, USA; 3 Lead contact

## Abstract

Reproduction is an energy-intensive process requiring systemic coordination. However, the inter-organ signaling mechanisms that relay nutrient status to modulate reproductive output are poorly understood. Here, we use *Drosophila melanogaster* as a model to establish the integrated stress response (ISR) transcription factor, Atf4, as a fat tissue metabolic sensor that instructs oogenesis. We demonstrate that Atf4 regulates lipase activity to mediate yolk lipoprotein synthesis in the fat body. Depletion of *Atf4* in the fat body also blunts oogenesis recovery after amino acid deprivation and re-feeding, suggestive of a nutrient-sensing role for Atf4. We also discovered that Atf4 promotes secretion of a fat-body-derived neuropeptide, CNMamide, which modulates neural circuits that promote egg-laying behavior (ovulation). Thus, we posit that ISR signaling in fat tissue acts as a “metabolic sensor” that instructs female reproduction—directly by impacting yolk lipoprotein production and follicle maturation and systemically by regulating ovulation.

## In brief

Across phyla, female reproduction is intimately tied to nutrient availability. Here, Grmai et al. demonstrate that the evolutionarily conserved integrated stress response acts a nutrient sensor in *Drosophila* fat tissues to regulate female reproduction via two ways: by regulating yolk production and by modulating neurons responsible for egg laying.

## Graphical Abstract

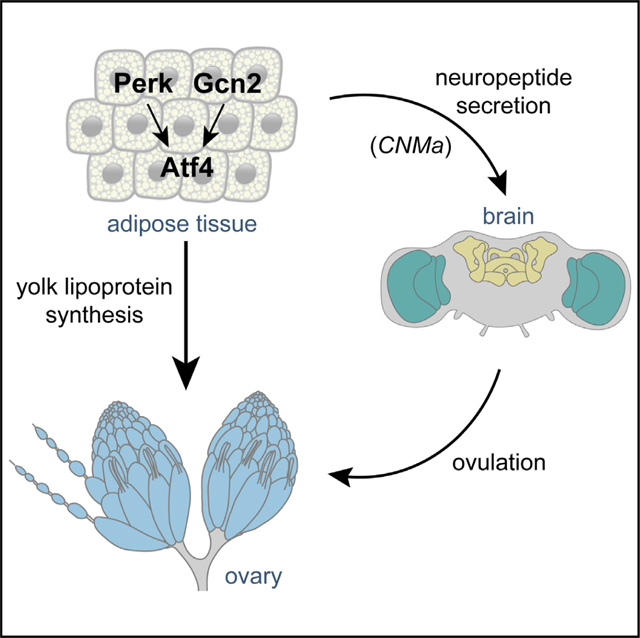

## INTRODUCTION

The fundamental behaviors of an organism, such as feeding and reproduction, are informed by its metabolic status. Reproduction relies on nutrient availability to support the high energetic cost of gametogenesis and associated reproductive behaviors. Across multicellular organisms, the fat tissue serves as a lipid storage organ and acts as a signaling hub to peripheral organs to communicate nutrient status.^1–3^ Consequently, fat tissue homeostasis broadly influences reproductive capacity; defects in such homeostasis due to insufficientor excess dietary lipids result in decreased fertility, especially in women.^4,5^ There is thus tremendous interest in understanding the molecular mechanisms that maintain fat tissue homeostasis and inter-organ signals that relay the loss of such homeostasis to peripheral organs, such as the ovary.

Highly metabolic tissues, such as fat and liver, have been reported to rely on stress response pathways to maintain homeostasis.^6–8^ Specifically, constitutive activity of the integrated stress response (ISR), an evolutionarily conserved pathway that relies on stress-sensing kinases, has been observed in adipocytes and hepatocytes.^7,9^ There are four known ISR kinases—heme-regulated eIF2a kinase (HRI, protein kinase R [PKR]), general control nonderepressible 2 (GCN2), and protein kinase R-like ER kinase (PERK)—that all signal through stress-response transcription factors when activated by endogenous or external stressors.^10^ The best-studied transcription factor downstream of the ISR kinases is activating transcription factor 4 (ATF4).^10^ The importance of ISR signaling in metabolic tissues is highlighted by patient mutations in ISR effectors and loss-of-function studies in model organisms. PERK mutations in humans cause Wolcott-Rallison syndrome, characterized by neonatal diabetes,^11,12^ and loss of PERK signaling in mouse models results in dysregulated liver glycogen content and increased hepatocyte death in high-sugar dietary conditions.^13,14^ In mice and fruit flies (*Drosophila melanogaster*), *Atf4*-mutant animals show lower body fat content, and *ATF4*^−/−^ mice show greater resistance to fatty liver under high-dietary-intake conditions.^6^

We and others have shown PERK and GCN2 to be constitutively active in fat tissues in mice and in *Drosophila*.^9,15,16^ Similar to loss of *Atf4*, loss of *Perk* or *Gcn2* results in dysregulation of fatty acid homeostasis, which is further exacerbated by dietary restriction or excess lipid or sugar intake.^13,15^ While the primary effects of the loss of ISR factors on fat tissues have been well studied, our understanding of how ISR-mediated fat tissue homeostasis impacts peripheral tissue function has been limited. Here, we use the *Drosophila* model to investigate how ISR signaling in fat tissue (called the “fat body”) affects female reproduction. The fat body is a highly metabolic tissue comprised of multiple cell types that together execute vertebrate liver and adipose tissue functions, including fat storage, detoxification, and immune response.^3^ A number of recent studies have demonstrated multiple signaling pathways, including GCN2, in the fat body to be essential for non-autonomous regulation of germline stem cell numbers in the ovary (reviewed by Lin and Hsu^2^). Another prominent role of the female fat body is the synthesis and trafficking of yolk lipoprotein to maturing oocytes, which is critical for oocyte maturation.^17^

**Figure 1.**
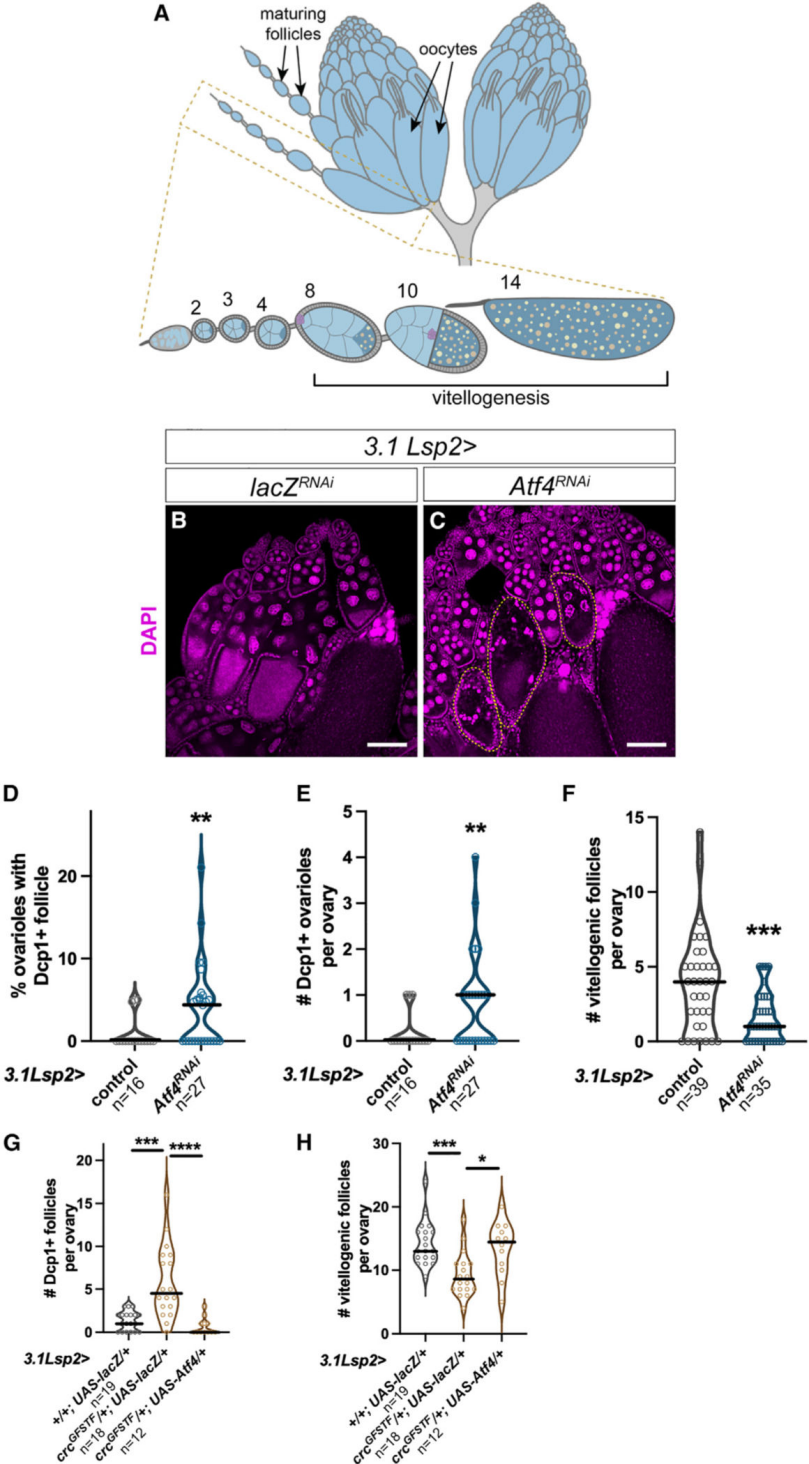
*Atf4* is required in the fat body for proper oogenesis (A) Schematic of adult *Drosophila* ovaries, showing clusters of ovarioles containing maturing follicles. The diagram at the bottom shows an individual ovariole with various follicle stages, with germ cells depicted in blue and somatic cells in gray. Vitellogenesis begins in stage 8, during which time yolk lipoproteins (orange spots) are trafficked from somatic cells into maturing oocytes (dark blue). Border cells (purple) are shown migrating in a stage 9 follicle and complete their migration at the anterior end of the stage 10 follicle. (B and C) Representative images of adult ovaries following control (*lacZ*, B) or *Atf4* (C) depletion from the fat body using *3.1Lsp2-GAL4*. Ovarioles marked with a dotted outline exhibit germ-cell nuclear fragmentation, indicative of dying follicles. DAPI marks nuclei in magenta. (D) Quantification of follicle death from (B) and (C) based on the percentage of ovarioles with a dying follicle (based on Dcp1 staining). (E) Quantification of follicle death from (B) and (C) as seen by cleaved caspase (Dcp1) staining. Death is quantified on the y axis as the number of ovarioles containing a dying follicle per ovary in each genotype. (F) Quantification of the rate of oogenesis from (B) and (C), reported as the number of vitellogenic follicles (stages 8–10) per ovary. (G and H) Quantification of rescue in follicle death (G) and oogenesis arrest (H) in *crc*^*GFSTF*^*/+* heterozygous females with ectopic Atf4 expression in fat tissues using *3.1Lsp2-GAL4*. Here and in following figures, the data are pooled from at least 10 animals collected across two biologically independent crosses; statistical analyses were performed using a two-tailed unpaired Student’s t test with Welch’s correction for unequal standard deviations. Asterisks indicate statistical significance as follows: *p < 0.05, **p < 0.01, ***p < 0.001, ****p < 0.0001; ns, not significant. The specific transgenic lines used in each experiment here and in the following figures are listed in Table S1.

The *Drosophila* ovary is organized as long chains of developing follicles called ovarioles^1^ (Figure 1A). Germline and somatic stem cells reside at the anterior apex and undergo differentiation along the ovariole in individual follicles. The germline stem cells differentiate to give rise to 16-cell germ cell “cysts,” with one designated to be the oocyte and 15 supporting nurse cells. Concomitantly differentiating somatic cells envelop the germ cyst to form a follicle. Each follicle undergoes 14 stages of oogenesis, culminating in a mature oocyte (Figure 1A). From stages 8–14, the oocyte accumulates yolk lipoprotein, which is synthesized both by follicle cells within the ovary and fat body surrounding the ovary.^18^ We have reported previously that fat-body-specific depletion of *Atf4*, encoded by *cryptocephal* (*crc*) in *Drosophila*, results in a decreased rate of oogenesis (termed oogenesis arrest previously and here), accompanied by follicle death.^19^ In this study, we determine the underlying mechanisms of these oogenesis defects.

## RESULTS

### Atf4 in the fat body non-autonomously regulates oogenesis

We have shown previously that global *Atf4*-mutant animals exhibit reduced egg laying and increased death of mid-oogenesis follicles.^19^ To determine whether these oogenesis defects were ovary autonomous, we had examined Atf4 expression in the ovary. We observed that an Atf4-GFP fusion protein encoded by a protein trap allele (*crc*^*GFSTF*^) was not detected in adult ovaries.^19^ We had utilized the GAL4/upstream activating sequence (UAS) binary expression system^20^ to deplete *Atf4* specifically in the fat body and observed that such loss was sufficient to recapitulate the reduced egg laying seen in *Atf4* mutants (Figure S5).^19^ To further characterize this phenotype, we sought to test whether the observed reduction in egg laying was due to the increased follicle death seen in *Atf4* mutants. We detected follicle death either by the presence of cleaved caspase staining (Dcp1 in *Drosophila*) or by observing germ cell nuclear fragmentation (discernible with DAPI staining), which is indicative of follicle death. We found that RNAi knockdown of *Atf4* using the fat-body-specific driver *3.1Lsp2-GAL4*^21^ (hereafter referred to as *3.1Lsp2>Atf4*^*RNAi*^) resulted in an increase in the number of Dcp1-positive follicles (Figures 1B and 1C). We observed a similar increase in follicle death upon *Atf4* knockdown using another driver, *Dcg-GAL4*, which is active in the fat body and the ovary (Figure S1A). To eliminate the possibility that there were ovary-autonomous effects for Atf4, we also tested ovary germline (*nos-GAL4*) and somatic (*tj-GAL4*) drivers and found no significant increase in follicle death (Figures S1B and S1C). Together, these data convincingly implicate a non-autonomous role for fat-body Atf4 signaling in the oogenesis defect observed in *crc*^*GFSTF*^ animals.

A prominent checkpoint in mid-oogenesis occurs at the onset of vitellogenesis (stage 8), during which maturing oocytes take up yolk proteins and lipids^18^ (Figure 1A). We observed that follicle death in *3.1Lsp2>Atf4*^*RNAi*^ was most frequent in stage 8–10 follicles, which are vitellogenic (Figures 1B and 1C). Such vitellogenic follicle death in *3.1Lsp2>Atf4*^*RNAi*^ is distinct from nurse cell death, which corresponds with nurse cell apoptosis, which is known to occur in stage 11–13 follicles^22^ (Figure S1D). The difference in vitellogenic follicle death between control and *3.1Lsp2>Atf4*^*RNAi*^ was statistically significant when quantified as the percentage of ovarioles containing at least one Dcp1-positive follicle (Figure 1D) or as the number of Dcp1-positive follicles per ovary (Figure 1E). In other words, we did not see a difference in the average number of ovarioles between control and *3.1Lsp2>Atf4*^*RNAi*^ animals (Figure S1E). Thus, for ease of data acquisition, we quantified further phenotypes on a per-ovary basis rather than on a per-ovariole basis.

In addition to increased follicle death, we also observed that *3.1Lsp2>Atf4*^*RNAi*^ animals had substantially fewer vitellogenic follicles (stages 8–10) per ovary, which is indicative of a defect in follicle maturation (Figures 1B, 1C, and 1F). To ensure that these defects were due to fat-body Atf4 signaling, we attempted rescuing the oogenesis defects in *crc*^*GFSTF*^ mutants by restoring Atf4 expression only in the fat body using *3.1Lsp2-GAL4*. In these experiments, we incidentally discovered that heterozygous *crc*^*GFSTF*^*/+* animals also showed increased vitellogenic follicle death and fewer vitellogenic follicles (Figures S1F–S1H, 1G, and 1H). While we were unable to recover *crc*^*GFSTF*^ homozygous animals with the *UAS-Atf4* rescue transgene, we did recover a small number of *crc*^*GFSTF*^/+ heterozygous animals with the *UAS-Atf4* rescue transgene. Analysis of these animals revealed that, indeed, restoring Atf4 expression in the fat tissues of Atf4 loss-of-function mutants was sufficient to rescue both vitellogenic follicle death and the number of vitellogenic follicles (Figures 1G and 1H).

Next, we asked whether the oogenesis defects in *3.1Lsp2>Atf4*^*RNAi*^ animals were the result of developmental contributions of Atf4. To do so, we utilized a *GAL80*^*TS*^ transgene alongside *3.1Lsp2-GAL4* (hereafter called 3.*1Lsp2*^*TS*^). In the presence of GAL80^TS^, rearing animals at a restrictive temperature (18°C) prevents GAL4 activity during development, while shifting adults to a permissive temperature (24°C) permits GAL4 activity. We did not observe a statistically significant increase in follicle death or oogenesis arrest upon adult-only *Atf4* depletion (Figures S1I and S1J), suggesting a developmental contribution for fat-body Atf4 in supporting oogenesis. Together, these oogenesis defects led us to pursue a non-autonomous role for fat-body Atf4 signaling in follicle maturation, which we further describe below.

### Atf4 promotes yolk lipoprotein secretion from the fat body

**Figure 2.**
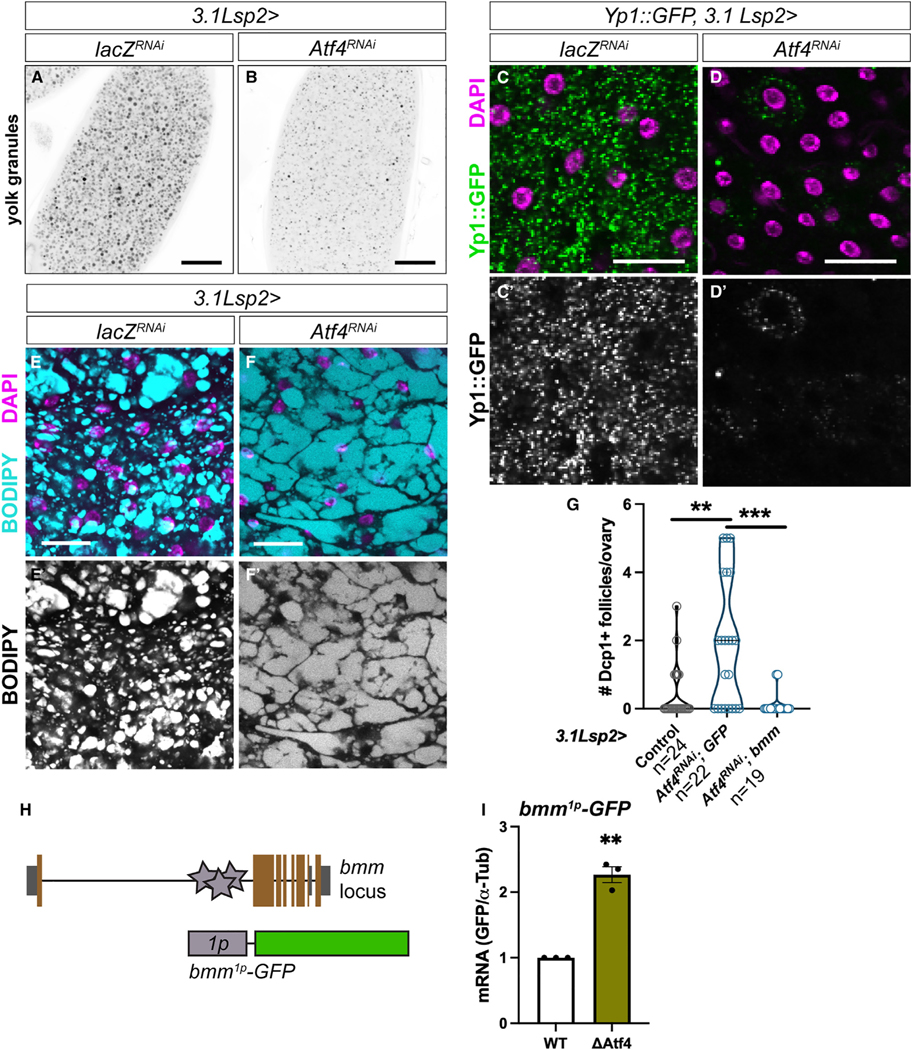
Atf4 activity in the fat body promotes vitellogenesis via yolk lipoprotein assembly in adipocytes (A and B) Representative confocal images of stage 14 oocytes from *3.1Lsp2>lacZ*^*RNAi*^ (A) and *>Atf4*^*RNAi*^ (B) females. Yolk granules were visualized by auto-fluorescence with a 405-nm laser; yolk granules appear as black/gray circles. Scale bars, 200 μm. (C and D) Representative confocal images of Yp1::GFP (green) expression in adult fat bodies from *3.1Lsp2>lacZ*^*RNAi*^ (C) and *Atf4*^*RNAi*^ (D) females. Scale bars, 50 μm. (E and F) Representative confocal images of neutral lipid staining (BODIPY, cyan) in adult fat bodies from *3.1Lsp2>lacZ*^*RNAi*^ (E) and *Atf4*^*RNAi*^ (F) females. Scale bars, 50 μm. (G) Quantification of follicle death in control ovaries compared with loss of *Atf4* (*3.1Lsp2>Atf4*^*RNAi*^*; >GFP*) and rescue by *bmm* co-expression (*3.1Lsp2>Atf4*^*RNAi*^*, >bmm*). (H) Schematic depicting Atf4 binding sites (gray stars) within the first intron of the bmm gene. A 385-bp region from this intron (*1p*) containing three Atf4 binding sites was cloned upstream of the GFP gene to regenerate the *bmm*^*1p*^*-GFP* reporter, which was used for S2 cell expression analysis in (I). (I) Relative GFP expression as determined by qPCR analysis in S2 cells transfected with wild type (WT) *bmm*^*1p*^*-GFP* and a mutant reporter lacking all three Atf4 binding sites (ΔAtf4, *bmm*^*1p-ΔAtf4*^*-GFP* in the text). In confocal images, DAPI (magenta) labels DNA.

Since we observed increased follicle death in vitellogenic stages, we examined whether loss of Atf4 signaling in the fat body affected yolk protein accumulation in maturing follicles. In *Drosophila*, yolk proteins are synthesized in both the somatic gonad and the abdominal fat body.^17^ We found that stage 14 oocytes from *3.1Lsp2>Atf4*^*RNAi*^ animals had substantially less yolk granule accumulation compared with controls (Figures 2A and 2B). Thus, we hypothesized that loss of ISR factors in the fat body compromises yolk protein trafficking to the ovary, which results in follicle death by disrupting vitellogenesis.

Yolk proteins are trafficked from the fat body to the ovary as lipoprotein vesicles, which contain a lipid core surrounded by the lipoproteins Yp1, Yp2, and Yp3.^23^ The lipid core of lipoproteins is derived from lipid droplets, which are extensions of the endoplasmic reticulum (ER) that bud off to form protein-coated vesicles.^24^ We tested whether there was an overall decrease in yolk lipoprotein production by visualizing expression of a yolk protein 1 (Yp1)-GFP fusion protein (Yp1::GFP)^25^ in the fat body. Knockdown of *Atf4* in the fat body showed a dramatic reduction of Yp1::GFP in adipocytes (Figures 2C and 2D), suggesting that loss of *Atf4* resulted in impaired yolk lipoprotein synthesis in the abdominal fat body.

Given the prominent role of Atf4 in lipid homeostasis,^6,26^ we next assayed for changes in lipid droplet formation in the fat body with loss of *Atf4*. Staining with the neutral lipid dye BODIPY showed that control fat bodies contained lipid droplets that characteristically organized into vesicles of varying sizes (Figures 2E, E′). In contrast, the BODIPY staining in *Atf4*-depleted fat bodies showed larger lipid droplets with a marked absence of discrete vesicular structures (Figures 2F and F′). We also observed similar large lipid droplets by BODIPY staining in fat bodies dissected from *crc*^*GFSTF*^ mutants (Figures S2A and S2B). Consequently, we reasoned that the BODIPY staining in *3.1Lsp2>Atf4*^*RNAi*^ abdominal fat bodies (Figures 2E and 2F) is indicative of aberrant lipid droplet formation, which consequently impacts yolk lipoprotein synthesis.

We next sought to determine the underlying mechanism by which Atf4 affects lipoprotein synthesis in the fat body. Lipoprotein assembly relies on mobilization of triglyceride (TAG) reserves in lipid droplets by lipases, such as the adipose triglyceride lipase (ATGL)-like *Drosophila* lipase Brummer (encoded by the gene *bmm*).^27^ Interestingly, fat tissues from *bmm* mutants also contain large lipid droplets,^27^ similar to what we observed in *3.1Lsp2>Atf4*^*RNAi*^ and *Atf4*-mutant animals (Figures S2C and S2D). Based on this evidence, we pursued *bmm* as a potential Atf4 target in the context of lipoprotein assembly. We used the publicly available ENCODE chromatin immunoprecipitation sequencing (ChIP-seq) data^28,29^ to assess Atf4 occupancy on the *bmm* locus. As a positive control, this dataset showed enrichment of Atf4 binding at the *Thor* locus, a known transcriptional target of Atf4^9^ (Figure S2E). A similar analysis showed several Atf4 binding events within the first intronic locus of *bmm* (Figure S2E). We next used the known position weight matrix for Atf4 to bioinformatically predict putative Atf4 binding sites, as described previously.^30^ Such analyses found several predicted Atf4 binding sites in the *bmm* intronic locus of varying strengths (Figure S2F). These observations prompted us to examine whether restoring *bmm* expression would be sufficient to rescue the follicle death seen with loss of *Atf4* in the fat body (Figures 1B and 1C). Indeed, we found that co-expressing *bmm* in the fat body reduced both the number of dying follicles in *3.1Lsp2>Atf4*^*RNAi*^ animals (Figure 2G) and lipid droplet size in adipocytes (Figures S2G–S2I).

Based on our bioinformatics analysis and rescue experiments above (Figures S2E and S2F, 2G), we hypothesized that *bmm* is likely a direct transcriptional target of Atf4 in the fat body. To test this hypothesis, we generated a GFP-based enhancer reporter using the *bmm* intronic locus where we found the Atf4 binding sites (*bmm*^*1p-WT*^*-GFP*; Figure 2H; see STAR Methods for details). We also generated a second similar reporter where the predicted Atf4 binding sites had been deleted (*bmm*^*1p-ΔAtf4*^*-GFP*). We expressed these reporters in S2 cells to determine via qPCR whether deleting Atf4 binding sites led to loss in GFP expression. Contrary to our prediction, we found that deleting Atf4 binding sites resulted in derepression of the *bmm1p* element, as seen by elevated GFP expression in the *bmm*^*1p-ΔAtf4*^*-GFP*-expressing cells in comparison with the *bmm*^*1p-WT*^*-GFP* expressing cells (Figure 2I). Consistent with the reporter data, qPCR analysis of fat bodies isolated from *3.1Lsp2>Atf4*^*RNAi*^ animals also showed increased *bmm* in comparison with control animals (Figure S2J). These surprising data led us to conclude that Atf4-mediated regulation of yolk lipoprotein synthesis in the fat body is not enacted by Bmm but by an unidentified lipase whose activity can be substituted for by Bmm, as substantiated by our rescue experiments.

### Atf4 in the fat body acts as a nutrient sensor to inform oogenesis

**Figure 3.**
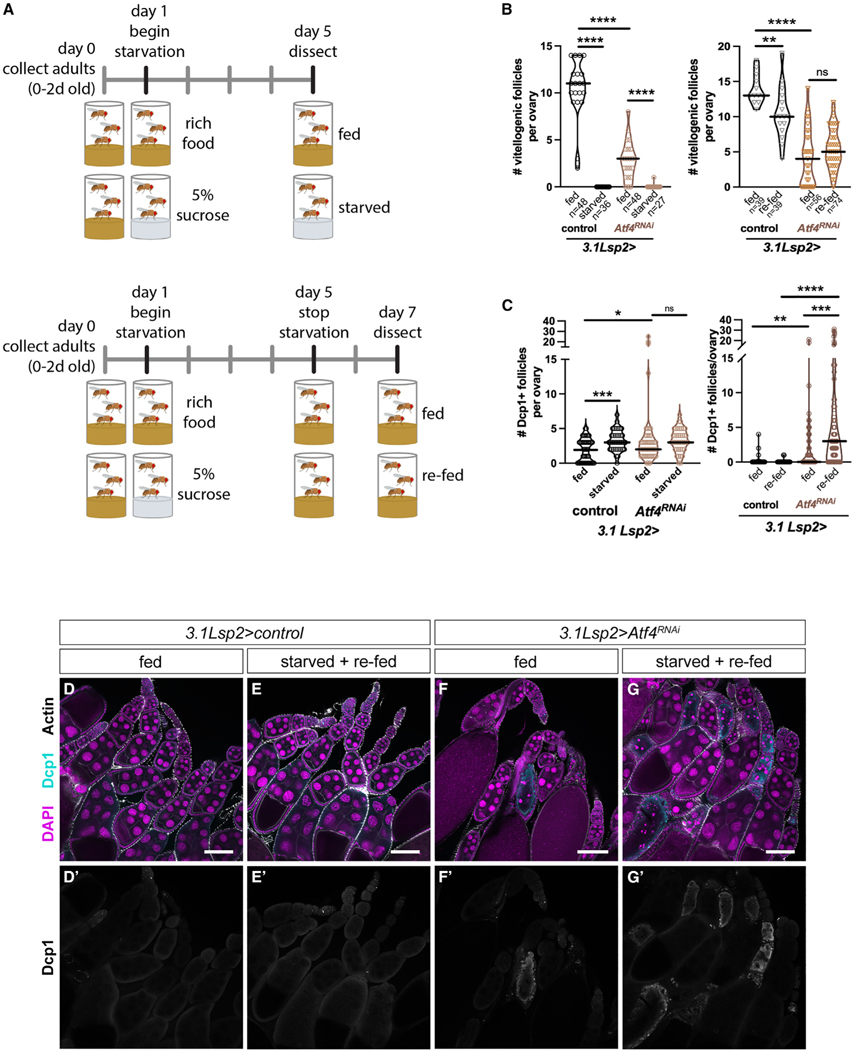
Atf4 is required for oogenesis recovery after amino acid deprivation (A) Diagram illustrating the starvation and re-feeding assay protocol. See STAR Methods and results for more details. (B) Quantification of vitellogenic follicles per ovary from control and *3.1Lsp2>Atf4*^*RNAi*^ females under fed, starved, or re-fed conditions. (C) Quantification of follicle death per ovary from control and *3.1Lsp2>Atf4*^*RNAi*^ females under fed, starved, or re-fed conditions. (D–G) Representative confocal images of ovaries from control (D and E) and *3.1Lsp2>Atf4*^*RNAi*^ (F and G) females under fed (D and F) or starved and re-fed (E and G) conditions. DNA is labeled with DAPI (magenta). Scale bars, 100 μm.

Nutrient deprivation has been demonstrated to induce oogenesis arrest and promote follicle death in a reversible manner.^31–33^ Nutrient deprivation, particularly amino acid deprivation, has also been documented extensively to induce Atf4.^9,19,34–37^ Thus, we asked whether oogenesis arrest during nutrient deprivation is mediated by Atf4. To do this, we performed a nutrient deprivation assay (Figure 3A, top), where mated females were raised for 4 days on 5% sucrose (starved) or nutrient-rich medium (fed). Notably, these dietary conditions have been independently shown to impact oogenesis^38^ and elevate Atf4 expression in the fat body.^9^ Since our data showed susceptibility to starvation in *Atf4* mutants after the 4-day time point, we elected to starve the animals for a maximum of 4 days. We measured the rate of oogenesis by quantifying the number of vitellogenic follicles per ovary. As expected, at the end of the starvation period, ovaries from “starved” females contained significantly fewer vitellogenic follicles compared with ovaries from “fed” females in control animals (Figure 3B). In addition, we saw a significant increase in vitellogenic follicle death in “starved” versus “fed” ovaries, as determined by Dcp1 staining (Figure 3C). We observed a similar increase in follicle death and oogenesis arrest in ovaries from starved versus fed *3.1Lsp2>Atf4*^*RNAi*^ females (Figure 3B).

The loss of maturing follicles induced by nutrient deprivation can typically be reversed by re-introduction of nutrients to the diet.^32^ Consistent with this, we found that re-feeding control females with nutrient-rich food following the 4-day starvation (Figure 3A, bottom) restored vitellogenic follicles to the ovary (Figure 3B). Additionally, we also found that the follicle death following starvation is substantially reduced after re-feeding in control animals (Figures 3C–3E). Analysis of ovaries from *3.1Lsp2>Atf4*^*RNAi*^ females showed resumption of oogenesis following starvation, as seen by the number of vitellogenic follicles (Figure 3B). However, we also saw a massive increase in the rate of death in both vitellogenic (stages 8–10) and pre-vitellogenic (stages 4–6) follicles in these ovaries after the re-feeding period (Figures 3C–3G), indicating impaired recovery of oogenesis after nutrient deprivation in *3.1Lsp2>Atf4*^*RNAi*^ females. Notably, there was no observable death in pre-vitellogenic follicles in control animals under these starvation conditions (Figure 3E). Such an increase in follicle death, particularly in pre-vitellogenic follicles, may be indicative of a sustained starvation response,^31^ suggesting that, in the absence of Atf4 in the fat body, female animals are unable to sense nutrient levels. Based on these data, we propose that Atf4 in the fat body is required for sensing changes in nutrient availability to regulate reproductive output.

### Fat-body ISR signaling independently modulates ovulation

**Figure 4.**
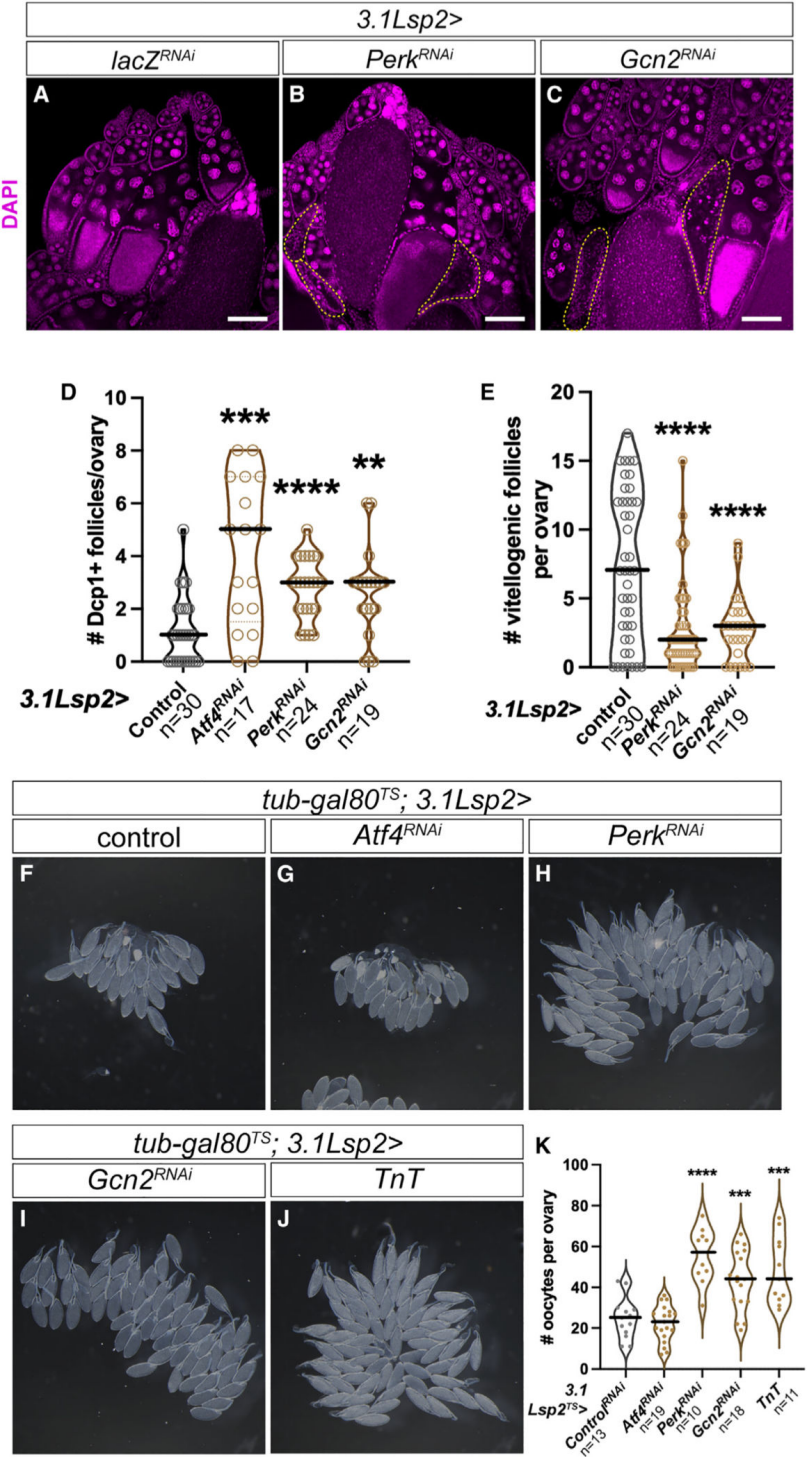
ISR signaling in the fat body modulates egg laying behavior (A–C) Representative ovaries from *3.1Lsp2*^*TS*^*> lacZ*^*RNAi*^ (A), >*Perk*^*RNAi*^ (B), and >*Gcn2*^*RNAi*^ (C) females. Dying follicles are encircled with a yellow dotted line, identified by nuclear breakdown. DAPI is shown in magenta. (D) Quantification of follicle death depicted in (A)–(C). (E) Quantification of vitellogenic follicles per ovary in (A)–(C). (F and J) Representative bright-field images of ovaries from *3.1Lsp2*^*TS*^*>lacZ*^*RNAi*^ (F), *Atf4*^*RNAi*^ (G), *Perk*^*RNAi*^ (H), *Gcn2*^*RNAi*^ (I), and *TnT* (J) females. Opaque oocytes located at the base of each ovary represent eggs retained in each ovary at the time of dissection. (K) Quantification of the egg retention phenotype shown in (F)–(J), reported as the number of stage 14 oocytes per ovary in the indicated genotypes.

There are two known ISR kinases upstream of Atf4 in *Drosophila*: the ER-stress sensor Perk and amino acid sensor Gcn2. To assess which of these kinases are required for Atf4 signaling in the fat body, we used previously validated RNAi lines to deplete *Perk* or *Gcn2*^9^ in the fat body using *3.1Lsp2-GAL4.* As we observed in *Atf4* knockdown animals, we found that knockdown of either *Perk* or *Gcn2* in the fat body resulted in increased death of vitellogenic follicles and a decrease in the number of vitellogenic follicles (Figures 4A–4E). Examination of maturing eggs from these animals also revealed a decrease in yolk granules as seen with loss of *Atf4* in the fat body (Figures S3A–S3C). These data suggest that both Perk and Gcn2 act upstream of Atf4 in the fat body.

Intriguingly, in addition to increased follicle death and mid-oogenesis arrest, we also observed a paradoxical accumulation of excess oocytes in *Perk* and *Gcn2* knockdown animals. These effects were observed even when *Perk* or *Gcn2* were knocked down only in adults, using *3.1Lsp2*^*TS*^ to suppress loss of ISR signaling in the fat body during development. Ovaries from mated females upon fat-body-specific depletion of *Perk* or *Gcn2* (*3.1Lsp2*^*TS*^*>Perk*^*RNAi*^ or *>Gcn2*^*RNAi*^) contained nearly twice as many mature oocytes per ovary (*Perk*^*RNAi*^: 55.4 ± 4.27, p < 0.0001; *Gcn2*^*RNAi*^: 43.56 ± 3.39, p < 0.001) than control ovaries (24.77 ± 2.78) (Figures 4F–4H, 4I, and 4K). Ovulation is exhibited in *Drosophila* by egg-laying behavior, and defects in this process result in retention of mature eggs in the ovary.^39^ While we did not observe this “egg retention” phenotype with *3.1Lsp2*^*TS*^*>Atf4*^*RNAi*^ (Figure 4G and 4K; 22.26 ± 2.00), we had previously reported a “swollen ovary” appearance in *Atf4* hypomorphic mutants.^19^ We reasoned that, since both Perk and Gcn2 signal to induce Atf4, loss of either kinase may result in weaker effects than loss of *Atf4*. This interpretation is supported by our observation that loss of *Perk* or *Gcn2* resulted in fewer Dcp1-positive follicles in comparison with loss of *Atf4* (Figure 4D). We further tested this by simultaneous depletion of *Perk* and *Gcn2* in the fat body. Indeed, *3.1Lsp2*^*TS*^*>Perk*^*RNAi*^*+Gcn2*^*RNAi*^ showed no egg retention phenotype (Figure S3D), similar to *3.1Lsp2*^*TS*^*>Atf4*^*RNAi*^ ovaries. Based on this, we conclude that substantial depletion of ISR signaling (by depleting the common downstream target Atf4) leads to severe oogenesis arrest. Such an arrest masks potential ovulation defects in these animals due to an overall decrease in maturing oocytes. Consequently, a role of ISR signaling in ovulation is only revealed with partial loss of function by depleting either upstream ISR kinase or by use of a hypomorphic *Atf4* allele.

Since we observed that ISR signaling regulates yolk lipoprotein secretion from the fat body to support oogenesis, we sought to examine whether other factors are similarly secreted from the fat body to regulate ovulation. To test this, we expressed tetanus toxin (TnT) in the fat body to block secretion mediated by vesicular exocytosis.^40^ Expression of *TnT* using *3.1Lsp2*^*TS*^*-GAL4* recapitulated the “egg retention” phenotype we observed with *3.1Lsp2*^*TS*^*>Perk*^*RNAi*^ or *>Gcn2*^*RNAi*^ compared with control animals (48.09 ± 4.94) (Figures 4J and 4K; 48.09 ± 4.94). Thus, in addition to regulating yolk lipoprotein synthesis, our genetic analysis revealed a new role of ISR signaling in ovulation, which we explore further below.

### ISR signaling in the fat body promotes ovulation via CNMamide (CNMa)-dependent activation of sexually dimorphic neurons

**Figure 5.**
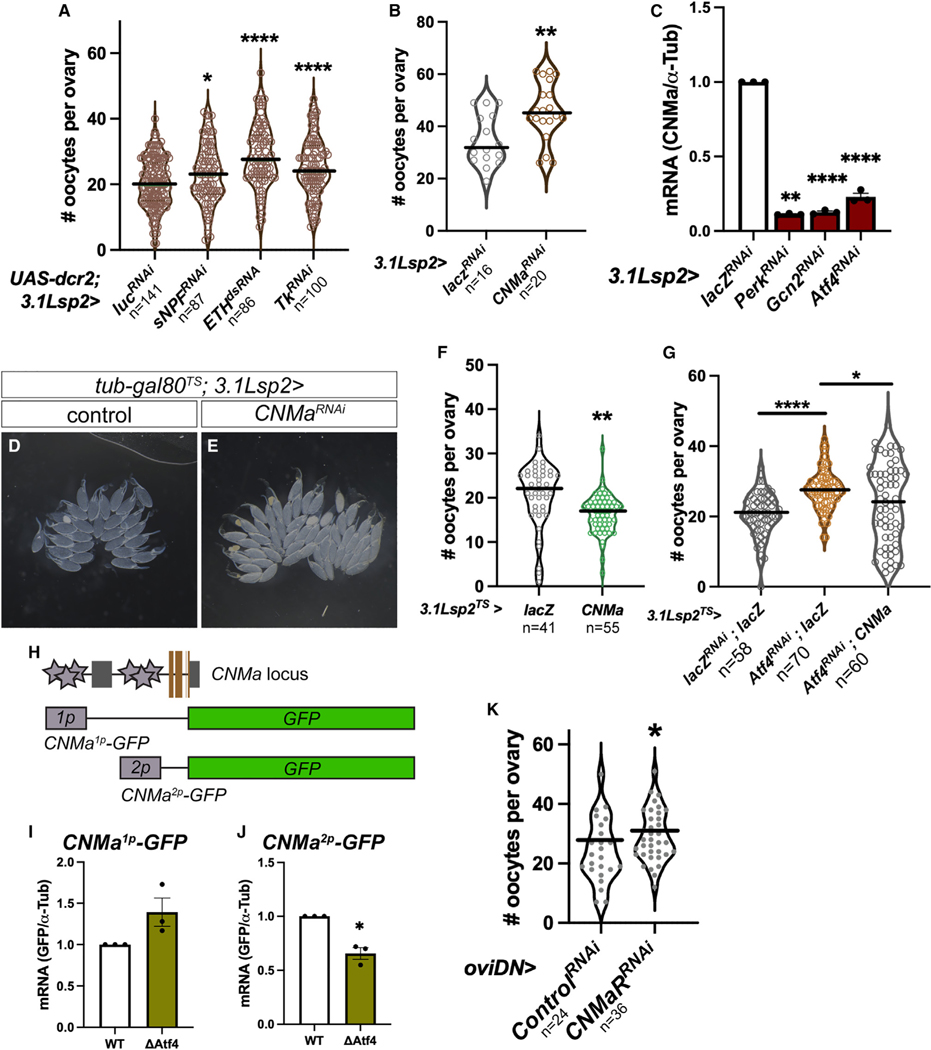
Oviposition-descending neurons (oviDNs) respond to ISR-regulated CNMa to promote ovulation (A and B) Quantification of oocytes contained per ovary in a limited neuropeptide screen (see Table S2 for a list), where putative Atf4-regulated neuropeptides were individually depleted by RNAi in the fat body. Please note that RNAi lines were obtained from multiple sources, necessitating different negative controls. (C) qPCR analysis of *CNMa* transcript abundance in fat bodies from control (*3.1Lsp2>lacZ*^*RNAi*^) versus *Perk*-, *Gcn2*-, or *Atf4*-depleted fat bodies. Values were normalized to *A-Tub84B* as a housekeeping gene. Values are reported as the average of three biological replicates. (D and E) Representative bright-field images of ovaries from *3.1Lsp2*^*TS*^*>control* (D) and *>CNMa*^*RNAi*^ (E) females. (F) Quantification of oocytes per ovary in control versus *3.1Lsp2*^*TS*^*>CNMa* females. (G) Quantification of the egg retention phenotype in fat bodies lacking Atf4 (*3.1Lsp2>Atf4*^*RNAi*^*; lacZ*) and those rescued with simultaneous expression of ectopic CNMa (*3.1Lsp2>Atf4*^*RNAi*^*; CNMa*). (H) Schematic depicting Atf4 binding sites (gray stars) within the promoter region and first intron of the *CNMa* gene. A 1,593-bp sequence from the promoter (*1p*) and a 2,486-bp sequence from the first intron (*2p*), each containing multiple Atf4 binding sites, were cloned upstream of the *GFP* gene to generate the *CNMa*^*1p*^*-GFP* and *CNMa*^*2p*^*-GFP* reporters, which were used for the S2 cell expression analysis in (I) and (J). (I and J) Relative GFP expression, as determined by qPCR analysis, in S2 cells transfected with WT *CNMa*^*1p*^*-GFP* (I) and *CNMa*^*2p*^*-GFP* (J) as well as mutant reporters lacking the contained Atf4 binding sites (ΔAtf4, *CNMa*^*1p/2p-ΔAtf4*^*-GFP* in the text). (K) Quantification of the egg retention phenotype in animals where the CNMa receptor (CNMaR) is depleted in sexually dimorphic neurons using a split *oviDN-GAL4.*

Our results thus far suggest that ISR signaling in the fat body promotes ovulation via a secreted factor. In *Drosophila*, ovulation is stimulated in mated females via activation of sexually dimorphic neural circuits.^41–45^ Thus, we hypothesized that the secreted factor downstream of ISR signaling is a neuropeptide that signals to subsets of these neurons to regulate ovulation. To identify putative Atf4-regulated neuropeptides, we compiled a comprehensive list of the annotated neuropeptides in the *D. melanogaster* genome based on FlyBase categorizations (Table S2). We next examined Atf4 occupancy in the genomic loci of these neuropeptides using the ENCODE ChIP-seq dataset described above.^28,29^ Analysis of the overlap between these two datasets revealed four neuropeptides with evidence of Atf4 occupancy (Table S2, highlighted in green). We systematically depleted each of these neuropeptides in the fat body using *3.1Lsp2-GAL4* and quantified egg retention (Figures 5A and 5B). We found that depletion of all four individual neuropeptides predicted to be Atf4 targets led to increased egg retention: *short neuropeptide F* (*sNPF*), *Ecdysis triggering hormone* (*Eth*), *Tachykinin* (*Tk*), and *CNMa* (Figures 5A and 5B). Further qPCR analysis using *Atf4* mutants showed a decrease in the mRNA abundance of two of these: *CNMa* and *Eth* (Figure S4A). *Tk* transcripts were undetectable by qPCR in both control and *Atf4* mutants, and publicly available data via FlyAtlas and modENCODE sequencing projects report no detectable *Tk* expression in larval or adult fat bodies. Of these, we found that fat-body-specific depletion of CNMa had the largest effect on egg retention, to similar extents as seen with *3.1Lsp2*^*TS*^*>Perk*^*RNAi*^ or *>Gcn2*^*RNAi*^ (Figures 5B and 5E; compare with Figures 4H, 4I, and 4K). Further, qPCR analysis of fat bodies from *3.1Lsp2*^*TS*^*>Perk*^*RNAi*^ or *>Gcn2*^*RNAi*^ animals also showed a substantial decrease in *CNMa* mRNA levels (Figure 5C), which prompted us to characterize the role of this neuropeptide in ovulation.

CNMa has been described previously to be induced in enterocytes of the gut in response to amino acid deprivation in an Atf4-dependent manner.^37^ Using a CNMa-GAL4 transgene,^37^ we found that the *CNMa* promoter is also active in both larval and adult female fat bodies (Figures S4B and S4C). Consistent with our prediction that CNMa is an Atf4 target, qPCR analysis showed lower levels of *CNMa* transcripts in *Atf4* mutants and in *3.1Lsp2>Atf4*^*RNAi*^ animals (Figures S4A, 5C). While CNMa depletion in the fat body led to egg retention (Figures 5B, 5D, and 5E), we found that overexpression of CNMa in the fat body conversely caused a reduction in oocyte number per ovary (Figure 5F). Based on this, we performed a rescue experiment to determine whether ectopic expression of CNMa can rescue the ovulation defects in *3.1Lsp2*^*TS*^*>Perk*^*RNAi*^ or *>Gcn2*^*RNAi*^ animals. However, we discovered that “diluting” GAL4 activity on the *UAS-Perk*^*RNAi*^*/Gcn2*^*RNAi*^ transgenes with a second *UAS* element (such as *UAS-lacZ* or *UAS-*CNMa) resulted in no detectable decrease in ovulation between control and *Perk*^*RNAi*^*/Gcn2*^*RNAi*^ animals (Fig. S4D). However, diluting GAL4 activity on the *UAS-Atf4*^*RNAi*^ transgene with a second *UAS* element now resulted in an apparent ovulation defect that was rescued by restoring CNMa expression in the fat body (Figure 5G). Together, these data substantiate a dose-dependent role of ISR signaling in ovulation via regulation of CNMa.

We next wanted to test whether CNMa is a direct transcriptional target of Atf4. To do so, we employed the same strategy as we did with our analysis with *bmm* (Figures 2H, 2I, S2E, and S2F). modENCODE analyses showed Atf4 occupancy in the promoter and within the first intron of the *CNMa* locus (Figure S2E). Thus, we used the position weight matrix of Atf4 to determine whether there were putative Atf4 binding sites within the promoter and first intron of the *CNMa* locus (*CNMa*^*1p*^ and *CNMa*^*2p*^, respectively). Our analysis revealed several predicted Atf4 binding sites in both *CNMa* elements (Figures S5A and S5B); from each of these elements, we generated GFP-based enhancer reporters (Figure 5H) similar to our construction of *bmm*^*1p*^*-GFP* reporters in Figure 2H. We transfected the resulting reporters, *CNMa*^*1p-WT*^*-GFP*, *CNMa*^*1p-ΔAtf4*^*-GFP*, *CNMa*^*2p-WT*^*-GFP*, and *CNMa*^*2p-ΔAtf4*^*-GFP*, into S2 cells and measured reporter activity by qPCR. These data revealed that, while deletion of the Atf4 binding sites in the *CNMa*^*1p*^ element had no significant effect on reporter activity (Figure 5I), the activity of the intronic *CNMa*^*2p*^ element was reduced by ~40% when the Atf4 binding sites were deleted (Figure 5J). Together, our qPCR analysis from fat bodies (Figure 5C) and enhancer element analysis (Figures S2E, S5A, S5B, and 5H–5J) submit that Atf4 likely directly targets the *CNMa* locus in the fat body.

Finally, we sought to identify which neurons respond to CNMa to promote egg-laying behavior. In *Drosophila*, egg-laying behavior is controlled by several groups of neurons, including two pairs of neurons descending from the mushroom body called oviposition descending neurons (oviDNs).^41^ The oviDNs are a subset of sexually dimorphic neurons in which the *P1* promoter of *fruitless* (*fru*^*P1*^) is active.^41^ Synaptic silencing of all *fru*^*P1*^-expressing cells blocks ovulation, as does ablation of oviDNs.^41^ We tested whether the CNMa receptor, CNMaR, is broadly required in all *fru*^*P1*^-expressing neurons; however, RNAi depletion of *CNMaR* using *fru*^*P1*^*-GAL4* resulted in an apparent block in oogenesis (Figures S5C–S5E), which likely would obscure an egg-retention phenotype. To circumvent this, we next depleted CNMaR specifically in the oviDNs using a *split-GAL4* driver combination^41^ (*oviDN-GAL4*). Consistent with our hypothesis that CNMa from the fat body signals to neurons that promote ovulation, we saw that RNAi-mediated depletion of *CNMaR* using *oviDN*-*GAL4* resulted in an increased number of eggs retained per ovary compared with control animals (Figure 5K; 29.19 ± 1.45 in *oviDN>CNMaR*^*RNAi*^ vs. 24.17 ± 2.16 in control, p < 0.05). Taken together, these findings support a model where ISR signaling in the fat body induces CNMa production, which is secreted to activate ovulation-promoting neurons such as the oviDNs.

## DISCUSSION

Single-celled organisms like yeast, where Atf4 (Gcn4 in yeast) was first discovered,^34,46^ rely on the ISR pathway under conditions of nutrient deprivation rather than for survival. In contrast, metabolically active tissues in higher organisms have evolved to rely on the ISR pathway for their homeostatic function.^47^ This is illustrated both by constitutive activity of Atf4 in fat tissues and by the metabolic phenotypes seen in *Atf4*-mutant *Drosophila* and mice.^6,9^ An increasing body of literature supports a role of fat tissues in modulating peripheral organ function,^48^ but whether and how this is regulated by ISR signaling is under active investigation. Our study makes a significant dent in this open problem by demonstrating multiple mechanisms by which homeostatic ISR signaling informs oogenesis in female flies.

**Figure 6.**
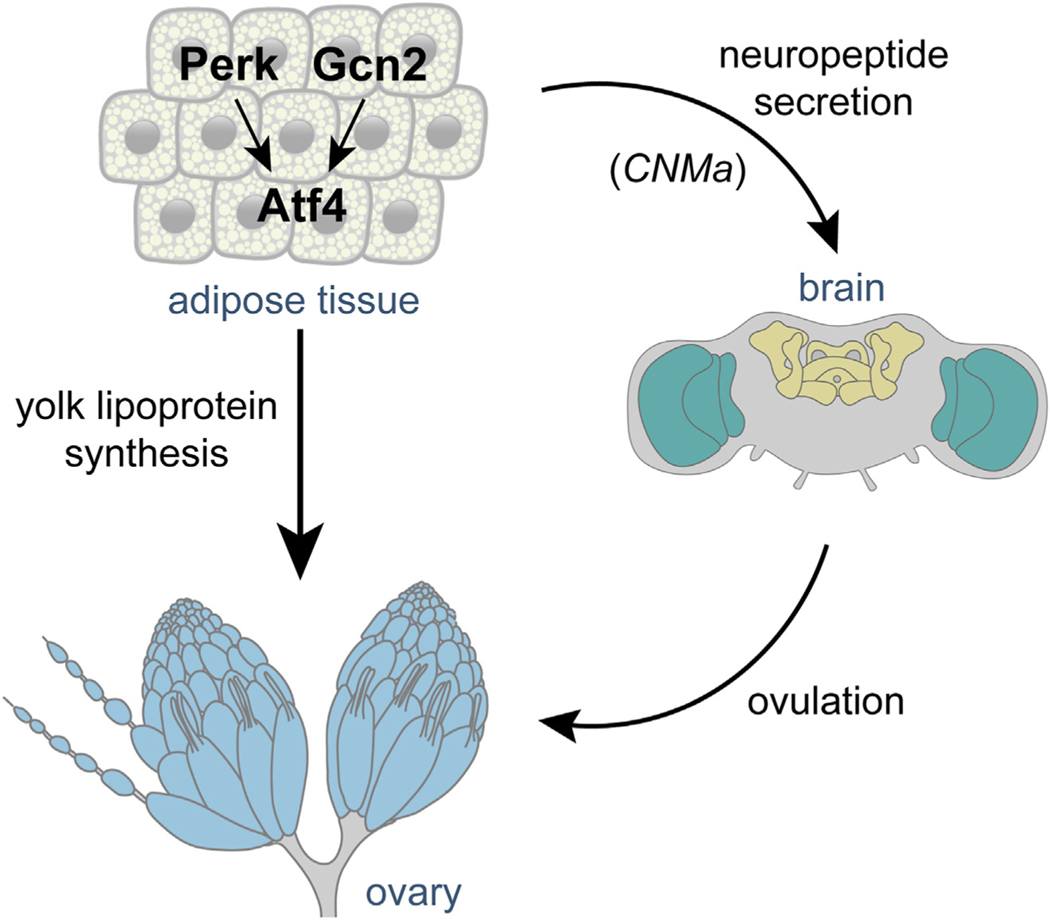
Model describing a role for ISR signaling in inter-organ regulation of reproduction.

Our findings show a requirement for both known ISR kinases, Perk and Gcn2, in both oogenesis and ovulation. Given that Perk is sensitive to changes in lipid content,^49^ and Gcn2 senses amino acid content,^50^ we favor a model where ISR signaling in the fat body acts as a conduit between nutrient status and reproductive capacity (Figure 6). In one branch of this model, oocyte maturation is compromised in the absence of Atf4 due to reduced yolk lipoprotein trafficking from the fat body to the ovary. In another seemingly parallel branch of this model, ISR signaling regulates ovulation, which is farther downstream of oogenesis, by neuromodulation of sexually dimorphic neurons. The sum effect of these two regulatory branches likely leads to the follicle death and oogenesis arrest observed in ISR-deficient animals and manifests as an overall decrease in fertility seen in global *Atf4* mutants.^19^ However, the phenotypes reported here may only be part of the effects of ISR signaling on oogenesis since a previous study demonstrated that fat body Gcn2 signaling impacts germline stem cell maintenance under low-amino-acid conditions.^51^ Together, these findings implicate ISR signaling in parallel processes with synergistic effects on female reproductive output.

### ISR signaling in the fat body is required for follicles to pass the vitellogenesis checkpoint

During oogenesis, maturing follicles are subjected to at least two checkpoints: the first is a meiotic checkpoint in the germarium in stage 2A, and the second is a mid-oogenesis checkpoint around stage 8.^52^ We found that *3.1Lsp2>Atf4*^*RNAi*^ animals largely showed death in vitellogenic follicles (Figure 1D), suggesting that these follicles were failing the latter mid-oogenesis checkpoint. This checkpoint is also considered a “nutritional checkpoint,” and previous work has demonstrated that follicles fail to progress beyond mid-oogenesis when lipid and/or protein is scarce.^31,53^ The fat body is thought to contribute approximately half of the yolk lipoprotein that is accumulated in the oocyte between stages 6 and 10.^17^ Thus, our finding that *3.1Lsp2>Atf4*^*RNAi*^ follicles die or arrest at the mid-oogenesis checkpoint is consistent with our observation of decreased yolk lipoprotein abundance in both adipocytes and oocytes from *3.1Lsp2>Atf4*^*RNAi*^ animals (Figures 2A and 2B). The importance of yolk lipoprotein synthesized and trafficked from the fat body to the oocyte is further underscored by our preliminary data showing that depleting *Yp1* in the fat body is sufficient to cause increased death in vitellogenic follicles (Figure S2K).

Yolk lipoprotein synthesis in the fat body is a multivariate process that relies on faithful formation of lipid droplets, which are then harvested by lipases to form lipoproteins. These particles are composed of a lipid core that is coated with yolk proteins, such as Yp1–3. Our data show that *3.1Lsp2>Atf4*^*RNAi*^ adipocytes have both aberrant lipid droplet formation (Figures 2E and 2F) and reduced levels of Yp1 protein (Figures 2C and 2D). Lipid droplets in *3.1Lsp2>Atf4*^*RNAi*^ animals appeared larger in size, which is consistent with what is seen in the absence of Bmm activity.^27^ However, our Atf4 binding site and qPCR analyses showed that *bmm* is likely not induced by Atf4 under homeostasis (Figures 2H, 2I, S2E, S2F, and S2J). Nonetheless, we found that ectopic *bmm* expression in *3.1Lsp2>Atf4*^*RNAi*^ animals rescued the vitellogenic follicle death phenotype and aberrant lipid droplet formation (Figures 2G and S2G–S2I), indicating that ectopic Bmm is likely substituting for another Atf4-regulated lipase. It also remains possible that Atf4 may regulate lipid droplet formation in additional ways other than lipase regulation. This possibility is supported by our preliminary data, where we see marked structural differences in the ER between control and *3.1Lsp2>Atf4*^*RNAi*^ animals (Figures S6A and S6B). That the ER is the primary site of lipid droplet biogenesis and that Atf4 is known to regulate fat content^6,26,54^ further lends credibility to the possibility that Atf4 impacts lipid droplet formation via multiple primary and secondary effects.

Our finding that *3.1Lsp2>Atf4*^*RNAi*^ and *Atf4* mutant adipocytes have larger lipid droplets (Figures 2E and 2F) is somewhat contrary to previous studies demonstrating that *Drosophila Atf4* mutants have lower overall TAG levels.^6,26^ However, consistent with these previous reports, our analysis also found that *Atf4* mutants have lower overall TAG levels (Figure S6C) but nonetheless show an increase in lipid droplet size within adipocytes (Figure S2A). Together, these data lead us to consider that the lower TAG levels in *Atf4* mutants may not be due to lipid storage defects in adipocytes but, rather, in another cell type. Fat bodies are comprised of two cell types: adipocytes, which are analogous to vertebrate adipose tissue, and oenocytes, which are analogous to vertebrate liver cells (hepatocytes). The decrease in TAG levels seen in *Atf4* knockout mice has been demonstrated to be specific to the liver,^26,54^ suggesting the possibility that Atf4 has different roles in adipocytes and hepatocytes. Such cell-type-specific roles are also supported by our preliminary data showing that fat bodies from *3.1Lsp2>Atf4*^*RNAi*^ animals show similar TAG levels compared with control animals (Figure S6D). An important implication of these findings is that TAG levels as determined by biochemical assays do not correlate well with lipid droplet size. Additionally, the possibility that the change in lipid organization and usage in *3.1Lsp2>Atf4*^*RNAi*^ animals impacts other peripheral organs, like muscles, that have independent roles in reproductive behaviors involving motility remains to be tested.

The dramatic reduction in Yp1::GFP abundance in fat seen with loss of *Atf4* (Figures 2C and 2D) is consistent with a defect in yolk lipoprotein synthesis but does not preclude the possibility that Atf4 may have effects on *Yp1–3* transcription or protein synthesis due to its aforementioned impact on ER structure (Figures S6A and S6B). Analysis of the modENCODE project^28,29^ ChIP-seq data revealed no Atf4 occupancy at the *Yp1*, *Yp2*, or *Yp3* locus (Figure S2B). Nonetheless, we observed a small but statistically significant decrease in *Yp1–3* mRNA levels in *3.1Lsp2>Atf4*^*RNAi*^ animals (Figure S2J), though it remains unclear whether this occurs via direct transcriptional regulation of *Yp1–3* by Atf4 or secondary effects of loss of *Atf4* in the fat body. In either case, there remains the possibility that loss of Yp1–3 gene expression contributes to defects in lipoprotein assembly independent of the TAG mobilization defects seen in *3.1Lsp2>Atf4*^*RNAi*^ animals.

### The role of ISR signaling in nutrient sensing

Atf4 is basally and constitutively active in the adult fat body,^6,9^ poising it to support reproduction under homeostatic conditions, as described above. However, Atf4 translation also increases in response to stress, notably amino acid deprivation, in the fat body and other tissues.^9,55^ Amino acid deprivation has a predictably negative impact on fertility, including a substantial increase in follicle death (paradoxically similar to loss of Atf4 in the fat) and eventual loss of germline stem cells (GSCs).^31,56,57^ How Atf4 signaling in the fat body differs between homeostatic vs. nutrient deprivation conditions remains an open question. Atf4 is a member of the ATF/CREB (activating transcription factor/cAMP response element binding protein) family of bZIP (basic leucine zipper) transcription factors with known interacting partners.^58,59^ Thus, an attractive possibility is that Atf4 regulates target gene expression in the fat body in concert with co-factors that change in abundance during starvation. It hence follows that, while Atf4 signaling is required for oogenesis during homeostasis,^19^ its homeostatic role would be mechanistically distinct from elevated Atf4 signaling under amino acid deprivation conditions. This distinction is supported by a previous study demonstrating that Gcn2 signaling in adipocytes mediates GSC loss under low-amino-acid conditions.^51^ However, the precise mechanism of how Gcn2 signaling implements GSC maintenance and the contribution of Perk signaling remain unknown.

Our data show that the increased vitellogenic follicle death and decreased rate of oogenesis seen with nutrient deprivation are not dependent on fat-body Atf4 signaling (Figure 3C). Strikingly, though, we saw increased death in not only vitellogenic but also pre-vitellogenic follicles even after re-feeding nutrient-deprived *3.1Lsp2>Atf4*^*RNAi*^ animals. We interpret these data as the *3.1Lsp2>Atf4*^*RNAi*^ animals failing to recognize that the organismal nutrient status had been restored, at least as it pertains to oogenesis. Thus, we posit that ISR signaling is a key metabolic sensor that passively permits or actively restricts oogenesis based on nutrient availability. In addition to Gcn2, amino acid levels are also sensed via the mTOR (mechanistic target of rapamycin) pathway,^60^ and similar to Gcn2, mTOR signaling in the adult fat body regulates GSC maintenance.^51,61^ Further, a vast body of literature demonstrates substantial interaction between Atf4 and mTOR signaling;^6,62–64^ Atf4 regulates expression of amino acid metabolism genes,^35^ and, consequently, *Atf4* mutant mice have reduced mTOR activity.^6^ Hypomorphic *Drosophila* mTOR mutants also present with small ovaries and elevated vitellogenic follicle death,^65^ indicating some level of redundancy between ISR and mTOR signaling in the fat body. These observations introduce the possibility that the effects of fat body Atf4 signaling on oogenesis could be compounded by an accompanying decrease in mTOR activity. Our preliminary data show that ectopic expression of mTOR in the fat body results in no significant rescue of follicle death (Figure S7A). However, we did observe a partial rescue of the vitellogenic follicle number in *3.1Lsp2>Atf4*^*RNAi*^ animals (Figure S7B). Ongoing work aims to precisely identify the molecular mechanisms by which Atf4 interacts with mTOR signaling in the fat body to regulate oogenesis under homeostatic and nutrient deprivation conditions.

While there is a clear emerging role for Gcn2 as an amino acid sensor in the fat body,^51^ the molecular role of Perk is less obvious. Perk is best known as a sensor for ER stress, which can be induced by misfolded proteins and lipid imbalance, among others.^10^ Perk has been convincingly demonstrated to be sensitive to membrane lipid saturation (via its transmembrane domain) in mammalian cells.^49^ Since *3.1Lsp2>Perk*^*RNAi*^ adipocytes contained fewer yolk lipoprotein particles (Figure S3B), an attractive hypothesis is that Perk acts as a lipid sensor in the fat body, thus complementing the amino-acid-sensing role of Gcn2.

### ISR signaling in the fat body promotes ovulation via CNMa secretion

Loss of either ISR kinase resulted in increased follicle death, decreased rate of oogenesis, and reduced yolk granules in maturing oocytes (Figures 4A–4E), demonstrating that both Perk and Gcn2 can act upstream of Atf4 in the adult fat body. Further, we observed that fat body knockdown of *Perk* or *Gcn2* resulted in retention of mature oocytes in the ovary (Figures 4H, 4I, and 4K), which is indicative of a defect in ovulation. We reason that depletion of either ISR kinase results in milder loss of Atf4, resulting in only a partial loss of ISR signaling, as opposed to *Atf4* knockdown, which results in greater loss of ISR signaling. Substantial loss of ISR signaling leads to a concomitant substantial oogenesis defect, thus obscuring any ovulation defects (Figures 4G and 4K). This notion is further supported by our data showing that joint knockdown of *Perk* and *Gcn2*, which leads to greater loss of ISR signaling in the fat body, does not display ovulation defects (Figure S3D). It is worth noting here that our data imply that proper ISR signaling in the developing fat body ensures yolk lipoprotein synthesis capacity in the adult (Figures S1I and S1J). However, ISR-mediated regulation of ovulation appears to be independent of a potential role of ISR signaling in fat body development (Figures 4F–4K).

Our data demonstrate that ISR mediates ovulation via multiple neuropeptides, including CNMa and Eth (Figures 5A and S4A), with CNMa being the largest contributor to ovulation. Based on our analysis, we propose that CNMa is likely directly transcriptionally regulated by Atf4 in the fat body (Figures 5C, 5H–5J, S2E, S5A, and S5B). The role of CNMa in the fat body is also supported by our finding that *CNMa* is constitutively expressed in larval and adult fat bodies under homeostatic conditions, similar to Atf4 (Figures S4B and S4C). Intriguingly, recent work has implicated CNMa in the amino acid deprivation response downstream of Gcn2 in enterocytes.^37^ However, this study utilized pan-neuronal drivers to demonstrate the role of the CNMa-CNMaR signaling axis in feeding behavior. In the context of ovulation, our data show that CNMa signaling via CNMaR appears to activate neurons, such as the oviDNs, that are known to promote egg-laying behavior.^41^ It is worth noting that CNMaR silencing specifically in oviDNs caused egg retention to a lesser degree than loss of CNMa from the fat body (Figure 5K). This leaves open the possibility that other cells (in addition to the oviDNs) may be responsive to CNMa in the context of egg-laying, including neurons in the central or peripheral nervous systems. For example, octopaminergic neurons in the brain and reproductive tract promote ovulation steps in response to mating-induced octopamine.^42^ Additionally, peripheral neurons that innervate the reproductive tract have been shown to regulate ovulation.^44^ High-throughput expression data generated by the modENCODE^28,29^ and FlyAtlas^66,67^ projects suggest that *CNMaR* expression is restricted to the head region, though it is possible that there are low levels of *CNMaR* in other cells. Further studies are needed to identify the entire cohort of cell types that are CNMa responsive in the regulation of ovulation downstream of ISR signaling in the fat.

### Limitations of the study

#### Developmental contribution of Atf4

We observed that adult-only knockdown of Atf4 in the fat body by introducing a temperature-sensitive GAL80 transgene (GAL80^TS^) did not lead to increased follicle death. This suggests a possible developmental role of Atf4 in fat body development. An alternate possibility is that shifting animal rearing from 18°C to 24°C for 3 days did not allow sufficient Atf4 depletion to observe defects in lipoprotein synthesis and follicle death. The commonly used “permissive” temperature for GAL80^TS^ is 29°C; we elected to upshift animals to 24°C instead of 29°C, as we observed severe fertility defects in females reared at 29°C.

#### Nutrient deprivation medium

It is worth noting that the dietary differences between our fed and starved animals extend beyond amino acids to carbohydrate composition, micronutrient availability, etc. Thus we remain open to the possibility that there may be additional nutrient-sensing mechanisms that may be at play for nutrient-deprivation-induced oogenesis arrest.

#### 3.1*Lsp2-GAL4 activity during starvation*

It has been reported previously that nutrient deprivation can affect *3.1Lsp2-GAL4* activity.^51^ When performing the starvation assays described here, we also found such reduced GAL4 activity, which may impact the extent of RNAi knockdown. We acknowledge that this may impact the strength of phenotypes pertaining to the role of Atf4 in mediating oogenesis arrest during starvation. However, the key phenotypes observed in these experiments are during oogenesis recovery after starvation, at which time GAL4 activity should be restored. During this time, we definitively see that oogenesis rate is not restored in the absence of Atf4 and, more so, observe pre-vitellogenic follicle death in these animals that was not observed anywhere else in the study.

#### Cell-type contaminants in adult fat body preps

In this study, we report qPCR and TAG data on adult fat body isolates. Since adult fat bodies are tightly associated with the inner abdominal wall, they are exceptionally hard to remove from surrounding tissue without substantial tissue loss. Thus, we present these data with the caveat that these sample preparations include other contaminating tissues, such as abdominal muscle, trachea, and dorsal vessel. Nevertheless, these crude preparations appear to be sufficient to observe known differences in lipid content between males and females^53,68^ (Figure S6D). Further, our genetic manipulations in these samples are driven by *3.1Lsp2-GAL4*, which is fat body specific and not active in any of the contaminating tissues, so we attribute the statistically significant changes reported here to loss of Atf4, Perk, or Gcn2 in the fat body.

#### GAL4 activity dilution with multiple UAS transgenes

The GAL4-UAS system is demonstrably sensitive to transgene dosage, with varying effects on the observed phenotypes depending on how many UAS binding sites are present per GAL4 transgene.^69–71^ This effect has somewhat limited our ability to perform certain rescue experiments in this study (e.g., Figure S4D), which we addressed using other complementary experiments that incidentally rely on the aforementioned dosage dependence (e.g., Figure 5G).

#### Effects of genetic background on number of vitellogenic follicles

In the course of compiling this study, the average number of vitellogenic follicles per ovary in control animals varied depending on the genetic background of the animals (e.g., control animals in Figure 1F vs. Figure 1H). Indeed, other studies have shown that there are substantial differences in stress response signaling across naturally occurring *D. melanogaster* genetic variants.^72,73^ Thus, though we controlled for genetic background whenever possible in this study, some of the differences we saw between control and loss-of-function animals may be due to unknown genetic background differences.

## STAR★METHODS

### RESOURCE AVAILABILITY

#### Lead contact

Further information and requests for resources and reagents should be directed to and will be fulfilled by the lead contact, Deepika Vasudevan (deepika.vasudevan@pitt.edu).

#### Materials availability

Plasmids are available upon request, fly strains used in this study are publicly available from the Bloomington Drosophila Stock Center or have been sourced from indicated individual laboratories (Table S1).

#### Data and code availability

- All data are available from the lead author upon request.
- This paper does not report original code.
- Any additional information required to reanalyze the data reported in this work paper is available from the lead contact upon request.

### EXPERIMENTAL MODEL AND STUDY PARTICIPANT DETAILS

#### Fly husbandry and stocks

The transgenic lines used in this study are publicly available via Bloomington *Drosophila* Stock Center or were sourced from other labs. See Table S1 for a complete list of lines used.

Animals were cultured in standard cornmeal agar media containing yeast and molasses (LabExpress, Inc). Fly stocks were maintained at either room temperature (RT) or 18°C and experimental crosses were maintained at 25°C. Adult females were collected at 0– 2 days following eclosion, mated with *w*^*1118*^ males for 3 days in standard media supplemented with yeast, and dissected after. For starvation assays, mated females were raised on amino acid-deficient medium containing 5% sucrose in 2% agarose for four days in the presence of *w*^*1118*^ males. To re-feed, females were transferred to fresh yeast-molasses medium vials for two more days. Conditional expression studies utilizing the *GAL80*^*TS*^ transgene were maintained at 18°C to restrict GAL4 activity during development, and 0–2 day adult progeny were shifted to 25°C for 3 days (with *w*^*1118*^ males) to permit GAL4-mediated knockdown and/or overexpression.

### METHOD DETAILS

#### Immunofluorescence

Adult ovaries and larval/adult fat body were dissected in PBS and fixed in 4% PFA for 15 min at RT. Samples were washed in PBS+0.1% detergent (Triton X-100 for ovary, Tween 20 for fat body) twice and incubated overnight at 4°C in primary antibody solution. Secondary antibodies along with DAPI (300nM final concentration) were incubated for 2 h at RT in the dark. Mouse anti-KDEL (Santa Cruz Biotech) was used at 1:100. Phalloidin-Rhodamine (Life Technologies) was used at 1:500. For yolk granule visualization, ovaries were fixed, washed, and mounted without DAPI. Yolk granules were visualized by auto-fluorescence upon excitation by 405nm laser.^74^ For neutral lipid staining in the adult fat body, samples were fixed and washed, followed by incubation in BODIPY solution (Thermo Scientific) for 20 min at a final concentration of 2 μg/mL. Confocal images were captured using a Nikon A1 confocal microscope through the Center for Biological Imaging at the University of Pittsburgh. Egg retention representative images were captured using a Nikon SMZ1270I with a ring light and DS-Ri2 camera attachment.

#### Fluorescent *in situ* hybridization (FISH)

FISH detection of *Atf4* mRNA was performed using a commercially available kit (Molecular Instruments, Inc) and was conducted as per manufacturer’s instructions.

#### ChIP-seq data analysis via ENCODE

ChIP-seq was performed on Atf4-GFP by Dr. Kevin White (UChicago) and is publicly accessible via the ENCODE^28,75^ project website (www.encodeproject.org). ENCODE accession number for dataset used: ENCFF986LGA. Briefly, embryos (0–22h) were isolated from *crc-MiMIC-GFP* transgenic fly line and ChIP was performed using an anti-GFP antibody with sequencing on the Illumina HiSeq 2000 platform. Reads were aligned to the dm6 *Drosophila melanogaster* genome annotation. Read frequency plots shown in Figure S2E were generated by loading ENCODE bigWig file onto the Integrated Genome Viewer software (www.igv.org; Broad Institute).

#### Quantitative RT-PCR

Abdominal adult fat bodies were dissected and after removing the ovaries, gut and other reproductive tissues, it was left attached to the carcass alongside muscles, pericardiocytes and oenocytes. RNA was extracted from four female adults per replicate using TRIzol (Invitrogen) according to manufacturer’s protocol. Reverse transcription was performed using Maxima H minus reverse transcriptase (Thermo Scientific) according to manufacturer’s protocol. qPCR amplification reactions were prepared using SYBR Green Master Mix (MidSci) in the BioRad CFX96 real time system. Table S3 lists primers used for analysis. All data are from at least 3 biologically independent replicates.

#### *Analysis of Atf4 binding sites in the* bmm *and* CNMa *locus*

We used a previously published Python code^30^ (https://github.com/finnroach/transcription-factor-binding) to determine if the *bmm1p*, *CNMa1p*, and *CNMa2p* elements have Atf4 binding sites. The entire first intron of *bmm* and *CNMa* was used for *bmm1p* and *CNMa2p* respectively. *CNMa1p* utilized the intergenic sequence preceding the *CNMa* transcription start site. To generate the GFP-based enhancer reporters, short “enhancer fragments” from within each region that included the predicted Atf4 binding sites were cloned into a modified version of pHStinger^76^ lacking the *Hsp70* promoter and containing attB recombination site. S2 cells were transfected with the reporters using Effectene (Qiagen) according to the DRSC protocol (https://fgr.hms.harvard.edu/stable-fly-cell-lines ) for 12-well format. After 48 h of transfection, RNA was extracted from cells using Trizol as described above.

### QUANTIFICATIONS AND STATISTICAL ANALYSIS

All quantifications represent data pooled from at least 10 animals collected across at least two biologically independent crosses. All graphs show data points from every animal examined. For egg retention quantifications, ovaries were dissected with minimal perturbance, fixed, washed, and mounted. The number of mature oocytes per ovary was then scored manually. The appropriate statistical analyses for each assay (indicated in the accompanying legend) were performed using Microsoft Excel or GraphPad Prism. All statistical analyses except qPCRs used Student’s t-test with Welch’s correction, which is appropriate for datasets with unequal variances and unequal sample sizes.

## Supplementary Material

1

2

## Figures and Tables

**Table:** KEY RESOURCES TABLE

REAGENT or RESOURCE	SOURCE	IDENTIFIER
Antibodies		

Mouse anti-KDEL (10C3)	Santa Cruz Biotech	Cat# sc-58774
Goat anti-mouse IgG (H + L) secondary, Alexa 488	ThermoFisher Scientific	Cat# A-11029
Phalloidin-Rhodamine	ThermoFisher Scientific	Cat# R415
BODIPY 493/503	ThermoFisher Scientific	Cat# D3922
Rabbit anti-Dcp1	Cell Signaling	Cat# 9578S
Chicken anti-GFP	Fisher scientific	Cat# 501962090

Bacterial and virus strains		

DH5Alpha	NEB	Cat #C2987H

Chemicals, peptides, and recombinant proteins		

Paraformaldehyde 16%	Electron Microscopy sciences	15710
Vectashield mounting medium	Vector Labs	H100010
Maxima H reverse transcriptase	Thermo Fisher	EP0753
PR1MA qMAX Green qPCR Mix	MidSci	PR2000-H-5000

Critical commercial assays		

Fluorescent *in situ* hybridization (FISH) kit	Molecular Instruments, Inc.	
Infinity Triglycerides Liquid Stable Reagent	ThermoFisher Scientific	Cat# TR22421
Effectene transfection reagent	Qiagen	Cat# 301425

Experimental models: Cell lines		

S2 cell line (*Drosophila melanogaster*)	Drosophila Genomics Resource Center	RRID:CVCL_TZ72

Experimental models: Organisms/strains		

See Table S1 for list of transgenic and mutant fly lines used.	This paper	N/A

Oligonucleotides		

See Table S3 for list of oligonucleotides used for qPCR analysis.	This paper	N/A

Recombinant DNA		

See File S1 for sequence of recombinant DNA	This paper	N/A

Software and algorithms		

Integrated Genome Viewer	Broad Institute	https://igv.org/doc/desktop/
Fiji-ImageJ	NIH	https://ImageJ.nih.gov/ij/

## Highlights

- Loss of ISR signaling components in fat tissues leads to premature follicle death
- ISR signaling regulates yolk lipoprotein production, which in turn impacts oocyte maturation
- The ISR transcription factor Atf4 directly regulates transcription of the neuropeptide CNMa
- CNMa regulates oviposition neurons; thus, loss of ISR signaling leads to ovulation defects
