# Supplementary material for: Integrated stress response signaling acts as a metabolic sensor in fat tissues to regulate oocyte maturation and ovulation
Source: Cell Rep. Author manuscript; Available in PMC 2024 May 8. (PMC11077669; doi:10.1016/j.celrep.2024.113863)
Supplement: 1 [file NIHMS1980919-supplement-1.pdf]

Cell Reports, Volume *43*

Supplemental information

Integrated stress response signaling acts as a metabolic sensor in fat tissues to regulate oocyte maturation and ovulation

Lydia Grmai, Manuel Michaca, Emily Lackner, Narayanan Nampoothiri V.P., and Deepika Vasudevan

Supplemental Information

Figures S1-S6 and Tables S1-S3

Integrated Stress Response signaling acts as a metabolic sensor in fat tissues to regulate oocyte maturation and ovulation

Lydia Grmai[1,2], Manuel Michaca[1], Emily Lackner[1], Narayanan Nampoothiri V.P.[1], Deepika Vasudevan[1,*]

[1]Dept. of Cell Biology, University of Pittsburgh School of Medicine, Pittsburgh, PA, USA

[2]Dept. of Psychiatry and Behavioral Sciences, Duke University Medical Center, Durham, NC, USA

*lead contact: deepika.vasudevan@pitt.edu

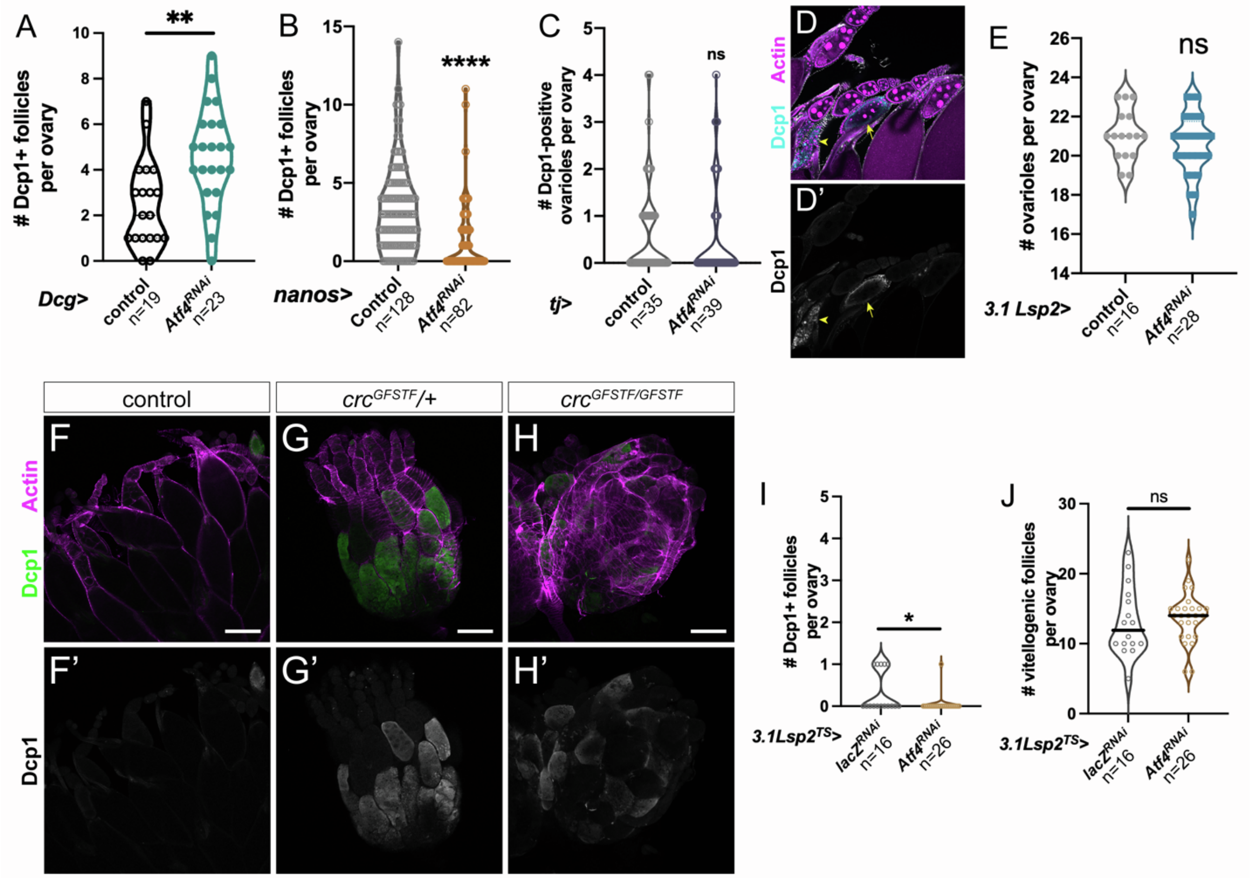

Figure S1. Atf4-mediated effects on oogenesis are tissue non-autonomous.

(A) Quantification of follicle death in *Dcg>Atf4*^{RNAi} ovaries compared with control ovaries (*Dcg>Atf4*^{RNAi}). Notably, *Dcg-GAL4* is expressed in both adipocytes and ovarian cells.

(B-C) Quantification of follicle death upon Atf4 knockdown using ovary drivers. Neither loss of Atf4 in germ cells using *nanos-GAL4* (B) nor loss in the somatic gonad using *tj-GAL4* (C) reproduced the increased follicle death observed in Atf4 mutants or upon fat body-specific Atf4 depletion.

(D) Representative image showing the distinction between developmental cell death in nurse cells versus vitellogenic follicle death. Representative ovary image shown is from a *3.1Lsp2>Atf4*^{RNAi} ovary. Dcp1-dependent nurse cell death was also seen in control ovaries and was not included in vitellogenic follicle death quantification for any genotype. Dcp1 is shown in cyan; DAPI is shown in magenta. Scale bar: 100 μm.

(E) Quantification of ovarioles per ovary in control versus *3.1Lsp2>Atf4*^{RNAi} ovaries compared with controls.

(F-H) Representative ovaries from control females (*3.1Lsp2>lacZ*), *Atf4* heterozygous mutants (*crc*^{GFSTF}*/+; 3.1Lsp2>lacZ*), and *Atf4* homozygous mutants (*crc*$^{GFSTF/GFSTF}$*; 3.1Lsp2>lacZ*). Images depict degree of follicle death and ovarian development in each genotype. Scale bars = 200 μm.

(I-J) Quantification of follicle death (I) and rate of vitellogenesis (J) in *3.1Lsp2*^{TS}>*Atf4*^{RNAi} ovaries compared with control ovaries.

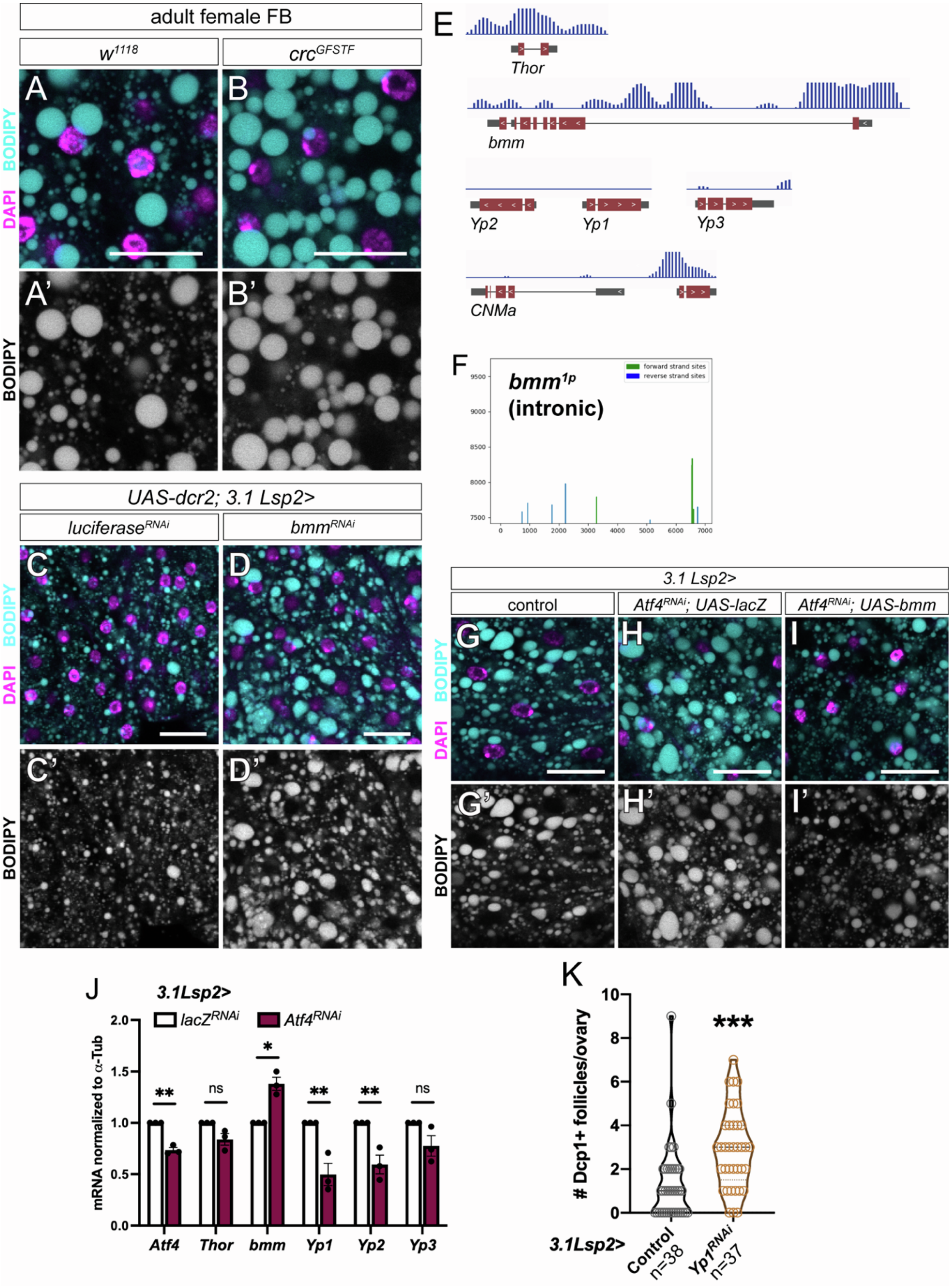

Figure S2. Atf4 mediates yolk lipoprotein production by regulating a lipase.

(A-B) Representative confocal images of neutral lipid staining (BODIPY, cyan) in adult fat body from control (*w^{1118}*, A) and *Atf4* hypomorphic mutant females (*crc^{GFSTF}*, B). Scale bar: 25 μm.
(C-D) Representative confocal images of neutral lipid staining (BODIPY, cyan) in adult fat body following control (*luciferase*, C) or *bmm* (D) depletion using the fat body-specific driver *3.1Lsp2-GAL4*. *UAS-dcr2* was added to both genotypes to improve the RNAi efficiency. Scale bar: 25 μm.
(E) Atf4 occupancy read abundance at specific gene loci, as determined by previously published ChIP-seq analysis of Atf4-GFP in *Drosophila* embryos[28,29]. In gene schematics, gray boxes represent UTRs, red boxes represent coding exons, and black lines represent introns.
(F) Results of *in silico* analysis of the *bmm* locus using a previously published Python code (see Methods). Results show multiple putative binding sites in the first intron of *bmm*.
(G-I) Representative confocal images of neutral lipid staining (BODIPY, cyan) in adult fat body from control (G), *3.1Lsp2>$Atf4^{RNAi}$; lacZ* (H), or *3.1$Lsp2^{RNAi}$; bmm* (I) females. Scale bar: 25 µm,
(J) qPCR analysis of transcript abundance in control (*3.1Lsp2>$lacZ^{RNAi}$*) versus Atf4-depleted (*3.1Lsp2>$Atf4^{RNAi}$*) fat bodies for indicated transcripts, normalized to *α-Tub84B* as a housekeeping gene. Values are reported as the average of three biological replicates.
(K) Quantification of follicle death following depletion of *Yp1* from the fat body using *3.1Lsp2-GAL4*.
In confocal images, DAPI labels nuclei in magenta.

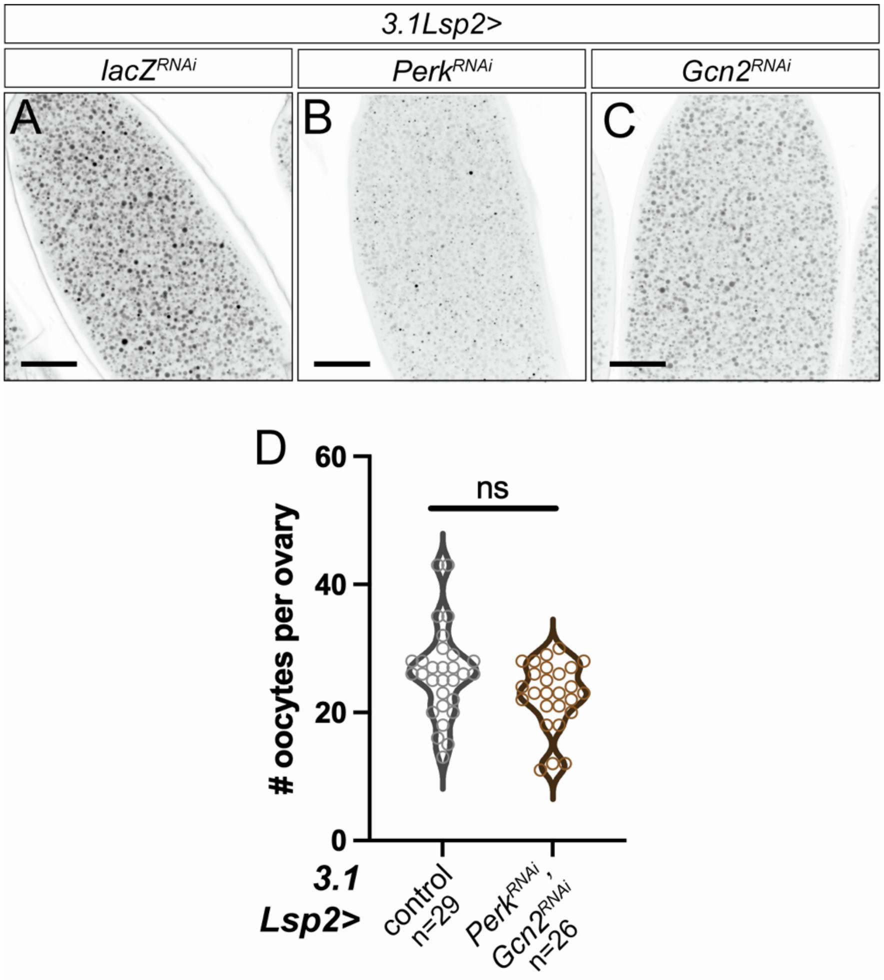

Figure S3. Loss of either ISR kinase in the fat body results in yolk granule defects in maturing oocytes.

(A-C) Representative confocal images of stage 14 oocytes from *3.1Lsp2>lacZ*^{RNAi} (A), *Perk*^{RNAi} (B), and *Gcn2*^{RNAi} (C) females using 405nm laser to visualize yolk granules. Scale bar: 200 μm.

(D) Quantification of oocytes per ovary upon double knockdown of *Perk* and *Gcn2* in fat tissue using *3.1Lsp2-GAL4* compared with *control*^{RNAi}.

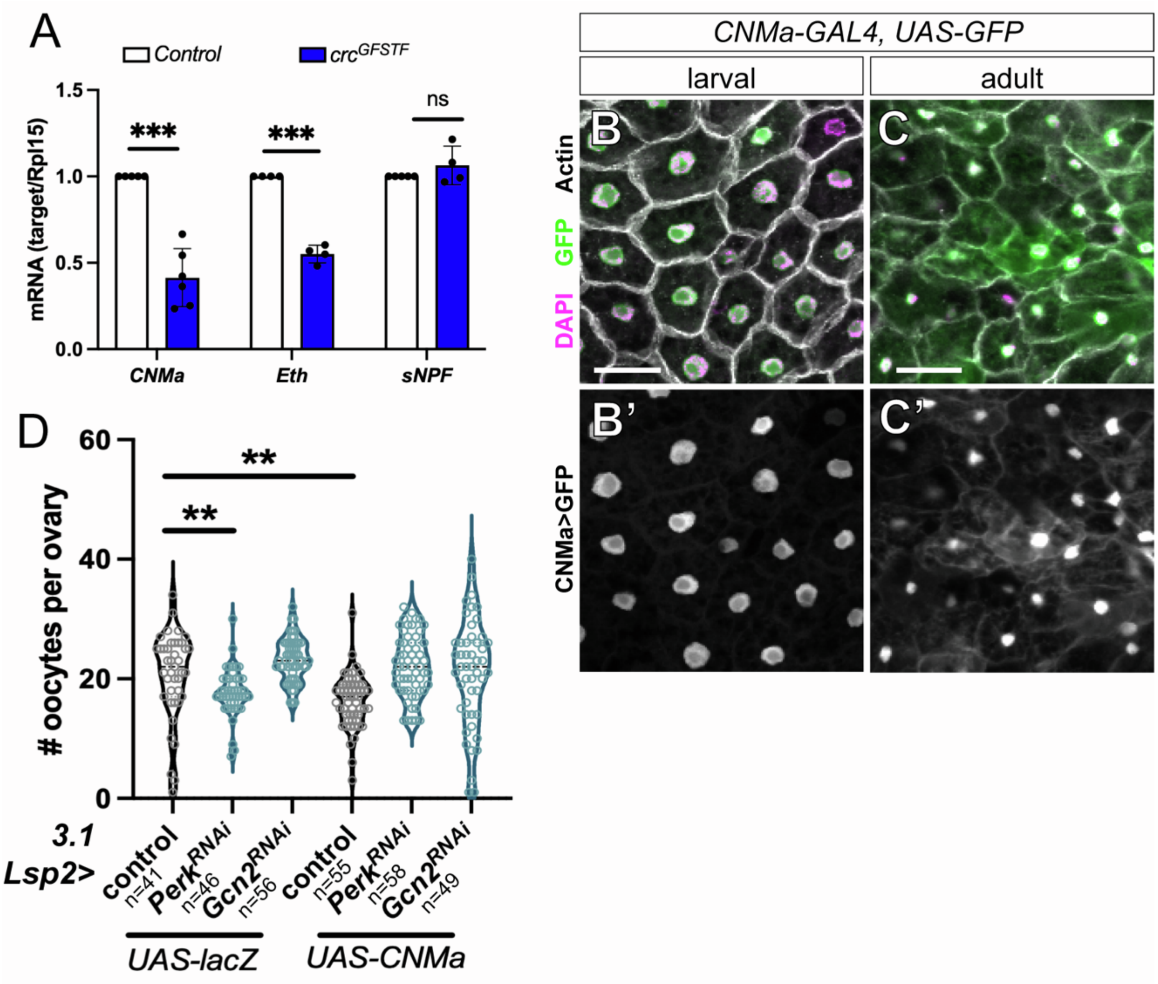

Figure S4. Atf4 regulates expression of CNMa in the fat body.

(A) qPCR analysis of neuropeptide transcript abundance in fat bodies from control (*yw*) versus *Atf4* hypomorphic mutants (*crc*^{GFSTF}) for *CNMa, Eth, and sNPF* normalized to *Rpl15* as a house-keeping gene. Values are reported as the average of at least four biological replicates. Please note that *Tk* mRNA could not be detected.

(B-C) Visualization of *CNMa-GAL4* driver expression in larval (C) and adult (D) fat tissues using *UAS-GFP*.

(D) Quantification of egg retention phenotype upon fat-specific depletion of *Perk* or *Gcn2* compared with control animals, in the presence of a second UAS construct – either UAS-lacZ or CNMa. Note that values for "control; *UAS-lacZ*" and "control; *UAS-CNMa*" animals are duplicated in **Fig. 5F**.

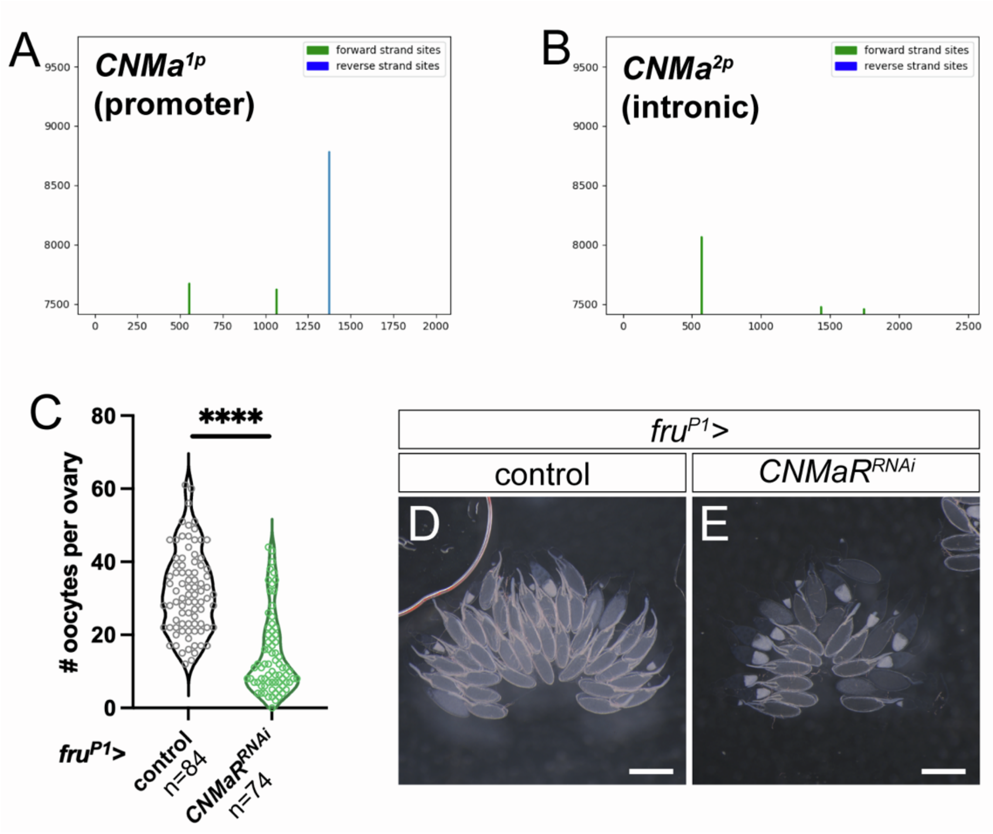

Figure S5. CNMaR is required in sexually dimorphic neurons for proper ovulation.
(A-B) Results of *in silico* analysis of the *CNMa* locus using a previously published Python code (see Methods). Results show multiple putative binding sites in the promoter (A, $CNMa^{1p}$) and first intron (B, $CNMa^{2p}$) of *CNMa*.
(C) Quantification of oocytes contained per ovary following control or *CNMaR* depletion in fru^{P1}-expressing cells. Black lines denote average for each genotype.
(D-E) Representative ovaries for C. Scale bar: 500 μm.

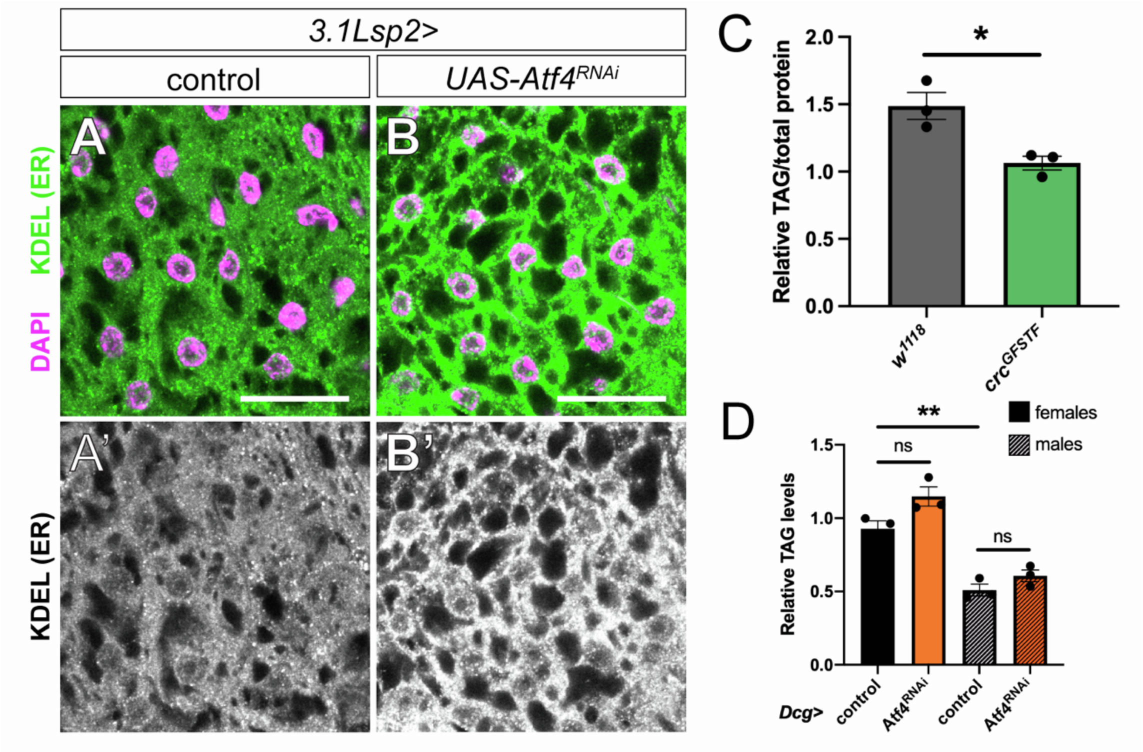

Figure S6. Loss of Atf4 in the fat body results in ER morphology and lipid storage defects.

(A-B) ER labeling of representative control (a) and *3.1Lsp2>Atf4^{RNAi}* (b) fat tissues using anti-KDEL antibody (green). DAPI (magenta) labels nuclei. Scale bar: 25 μm.

(C) Quantification of total triglyceride (TAG) levels normalized to total protein levels from control (*w^{1118}*) and Atf4 hypomorph (*crc^{GFSTF}*) adult females. Three females were used for each sample analyzed and values represent the average of three biological replicates.

(D) Quantification of total TAG levels normalized to total protein levels from crude fat body preparations from *3.1Lsp2>control* or *Atf4^{RNAi}* animals.

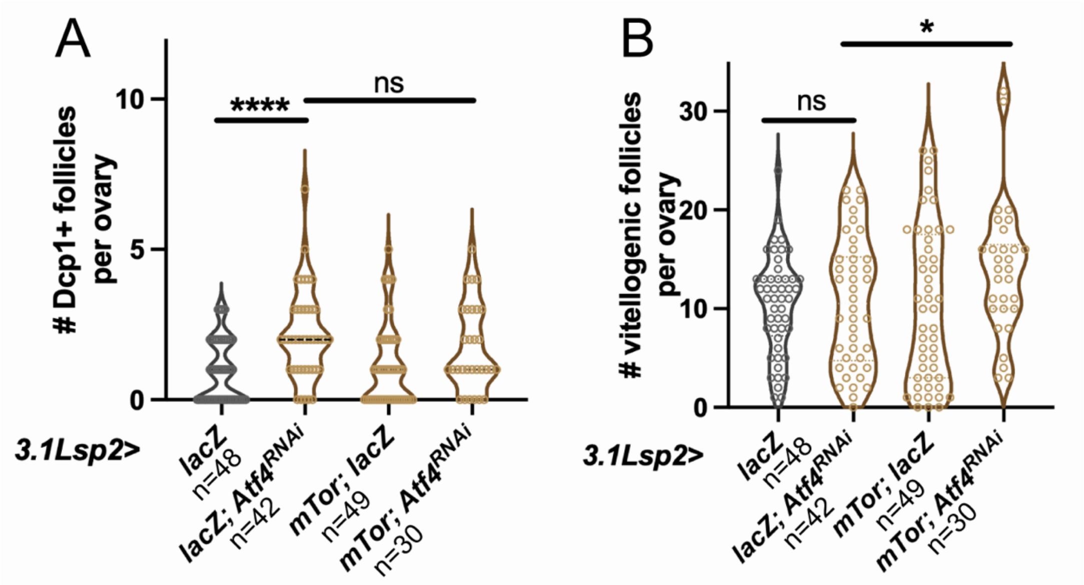

Figure S7. Ectopic expression of mTOR partially rescues the number of vitellogenic follicles caused by loss of Atf4 in the fat body.

(A-B) Quantification of follicle death (A) and rate of vitellogenesis (B) upon fat-specific Atf4 depletion and concomitant mTor expression. *UAS-lacZ* was used to control for UAS dosage.

Table S1. List of transgenic and mutant fly lines used in this study with stock numbers of source as indicated.

Genotype	Source	Figures used
P{GawB}tj^{NP1624} (tj-GAL4)	Kyoto #104055	S1C
nanos-gal4::VP16	BDSC #4937	S1B
3.1Lsp2-GAL4	BDSC #84285	1B-H; 2A-G; 3B-G; 4A-K; 5A-G; S1E,I,J; S2C-D,G-K; S3A-D; S4D; S6A-B; S7A-B
oviDN-GAL4	BDSC #86832	5K
fru^{P1}-GAL4	BDSC #66696	S5C-E
tub-GAL80^{TS}	BDSC #7019	4F-K; 5D-G; S1I-J
w^{1118}	BDSC #1495	S1F; S2A; S4A; S6C
crc^{GFSTF} (MI02300-GFSTF)	BDSC #59608	S1G-H; S2B; S4A; S6C
Yp1::GFP	Dr. Yusuke Hara[1]	2C-D
UAS-lacZ^{RNAi}	Dr. Hyung Don Ryoo[1]	1B-F; 2A-F; 4A,D,E,K; 5B-C,G,K; S1A, I, J; S2J; S3A,D
VIE-260B (control; isogenic background control for Atf4/Perk/Gcn2^{RNAi})	VDRC #106174	2G; 3B-E; S1B-C,E; S2G,K; S4D; S5D; S6D
UAS-luciferase^{RNAi}	BDSC #31603	5A; S2C
UAS-Atf4^{RNAi}	VDRC #109014	1C-F; 2B,D,F,G; 3B-C,F-G; 4D,G,K; 5C,G; S1A-C,E,I-J; S2H-J; S6B,D; S7A-B
UAS-Perk^{RNAi}	VDRC #110278	4B,D-E,H,K; 5C; S3B,D; S4D
UAS-Gcn2^{RNAi}	VDRC #103976	4C-E,I,K; 5C; S3C-D; S4D
UAS-CNMa^{RNAi}	VDRC #110796	5B,E
UAS-ETH^{dsRNA}	BDSC #26242	5A
UAS-sNPF^{RNAi}	BDSC #25867	5A
UAS-Tk^{RNAi}	BDSC #25800	5A
UAS-CNMaR^{RNAi}	VDRC #101076	5K; S5C,E
UAS-Yp1^{RNAi}	BDSC #67219	S2K
UAS-bmm^{RNAi}	BDSC #25926	S2D
UAS-dcr2	BDSC #24651	5A; S2C-D

UAS-bmm	BDSC #76600	2G, S2I
UAS-TeTxLC.tnt (UAS-TnT)	BDSC #28837	4J-K
UAS-lacZ	BDSC #3955 (ch.II); BDSC #3956 (ch.III)	1G-H; 5F-G; S2H; S7A-B
UAS-CNMa	Dr. Won-Jae Lee[1]	5F-G; S4D
UAS-Atf4	Dr. Hyung Don Ryoo[1]	1G-H
UAS-mTor	BDSC #53727	S7A-B
CNMa-GAL4.UAS-GFP	Dr. Won-Jae Lee[1]	S4B-C

Table S2. List of *Drosophila* neuropeptides and their gene symbols.
Candidates were included if they bore the "neuropeptide" classification on Flybase, and this list was compared to a previously curated list of neuropeptides[73] to verify that none were missing.

Neuropeptide gene	Symbol	Neuropeptide gene	Symbol
Adipokinetic hormone	*Akh*	*Insulin-like peptide 5*	*dilp5*
amnesiac	*amn*	*Insulin-like peptide 6*	*dilp6*
Allatostatin A	*AstA*	*Insulin-like peptide 7*	*dilp7*
Allatostatin C	*AstC*	*Insulin-like peptide 8*	*dilp8*
Allatostatin double C	*AstCC*	*ion transport peptide*	*ITP*
Bursicon	*Burs*	*Leucokinin*	*Lk*
Capability	*Capa*	*Myoinhibiting peptide precursor*	*Mip*
Crustacean cardioactive peptide	*CCAP*	*Myosuppressin*	*Ms*
CCHamide-1	*CCHa1*	*neuropeptide F*	*NPF*
CCHamide-2	*CCHa2*	*Neuropeptide-like precursor 1*	*Nplp1*
CNMamide[a]	*CNMa*	*Neuropeptide-like precursor 2*	*Nplp2*
Corazonin	*Crz*	*Neuropeptide-like precursor 3*	*Nplp3*
Diuretic hormone 31	*Dh31*	*Neuropeptide-like precursor 4*	*Nplp4*
Diuretic hormone 44	*Dh44*	*Orcokinin*	*Orcokinin*
Drosulfakinin	*Dsk*	*Partner of Bursicon*	*Pburs*
Ecdysis triggering hormone[a]	*Eth*	*Pigment-dispersing factor*	*Pdf*
Eclosion hormone	*Eh*	*Proctolin*	*Proc*
FMRFamide	*FMRFa*	*Prothoracicotropic hormone*	*PTTH*
Glycoprotein hormone alpha 2	*Gpa2*	*RYamide*	*RYa*
Glycoprotein hormone beta 5	*Gpb5*	*SIFamide*	*SIFa*
Hugin	*Hug*	*short neuropeptide F precursor*[a]	*sNPF*
Insulin-like peptide 1	*dilp1*	*Sex Peptide*	*SP*
Insulin-like peptide 2	*dilp2*	*space blanket*	*spab*
Insulin-like peptide 3	*dilp3*	*Tachykinin*[a]	*Tk*
Insulin-like peptide 4	*dilp4*		

[a]Four neuropeptides were identified as putative Atf4 targets based on ChIP-seq occupancy data via the ENCODE project (see Methods for details): *CNMa*, *Eth*, *sNPF*, and *Tk*.

Table S3. Primers used for qPCR analysis in this study are listed below.

Gene	Forward primer	Reverse primer
RpL15	AGGATGCACTTATGGCAAGC	CCGCAATCCAATACGAGTTC
Thor	CTTATCTACGAGCGGGCTTTC	AGGGAGTACGCGGAGTT
bmm	GTATGCACCGCATCTGTTGC	AGCGACGAGCTTCGTTCAC
Yp1	TTTCACCATTGAGCGTCTG	TCTTGTCACCATTGGGCT
Yp2	AAGCTCTACCATCTGTCCCAGT	CGCTTAACCTTCTCCACCAAAGT
Yp3	GAGAAGATCTACCACGTTGGC	GCACTCAACCTTCTGTCCAT
CNMa	GACCAATATGCCCAGCAAGA	GTAGGAATTGAAGCCAGCGT
Eth	TTCGCTCTTGGTGGGTCTTG	CAAAGTTCTCGCCTCGCTTG
sNPF	CTCGGCTCAAGGCACTC	GGATCACTGCGTCCGAATC
Tk	GCTGCAGGACTTCTTCGAT	CCTCCAGATCGCTCTTCTTG
GFP	GCA CAA GCT GGA GTA CAA CTA	TGT TGT GGC GGA TCT TGA A
α-Tub	CAA CCA GAT GGT CAA GTG CG	ACG TCC TTG GGC ACA AGA TC
