# Supplementary material for: Integrated stress response signaling acts as a metabolic sensor in fat tissues to regulate oocyte maturation and ovulation
Source: Cell Rep. Author manuscript; Available in PMC 2024 May 8. (PMC11077669; doi:10.1016/j.celrep.2024.113863)
Supplement: 2 [file NIHMS1980919-supplement-2.pdf]

List of sequences used in position frequency matrix analysis to identify Atf4 binding sites (in green)

>bmm_intron1
gtaagtctcttcccacttgcttacatgggtgggtctagagagggaagctttcggaatcattaagatctgccaactatatgatagcgattctc
caaggcgaaacaaaaataaattttttattttgctttgcataaatcttaatttgttaagtttcttgtttcttttcacactccgcgtctgaacttgg
cctttgcacaatgttttctgcttgggaaaacccctgtgaatattttctgcacccctctggtgttttctctactgctctcccctcttcgtgcagaa
tgcacacacacgaacacactcacacttgcgttcgcaaaacagctgagtgcaaaagcaaccacactgggcagttcagtgactctgtgagt
gggagtgagaaagagagcgagtgggagtgggagagcgcgaaagatgctgcgaggctcaatataaaacgcattgaattgaattttattg
ataagcttgtttgcgtttgtaggtcgctaggaagtcaatggggatctttcataattgactgcgatagtgtgtgtgtgtttttgggcgtgtttgt
ccaatttcgaagggggctcgtcccatccgctcaaaagaaaactgcggcgcagttgaaaaaccttacgaaaacagaaaaacaagtttcgt
atgcccgggacaacgcacttttgtaaagcggcacccgaatatatgggcaaatggttgggcacagcggtgggtatatgaatagcaacgca
gtccgaaaacatttcatcaaatacttttgcaaagagaataaactaaatacttatgctatgagattagccagaaactttataaatctaaatt
gaagataaaatcactcacagcatatcttatcttcaattgccaatattaaattaaatagattaaactggccttgtgatttagaattgcgatttc
ttttgcgaattgattaaagttaatttgcgtcagaatgataaagtcatatccttcatacaacctttatacaaaacctataaggcactataattc
tgaccgcaactgtatgcacacaaaacctattttgatcgagatgcttgaaggtcgagtgcttatttatacatatgtatatatgtatgtatattt
tgtttatgcttaggaggcgcggtcatcgaaagccgcctaggaatttgagtcaaaagtggtgccactgtggaatcgggtctatatataactg
cgaaatatatgcatacataatggacttgttcttgttgtcgattttcaaatgtaacgcttcgtttgtatcgtgggtataaacatgtcaatcactg
gggaactcaaaactagcgagggagcacaacaaatgtcagctggattactcatggctactgggctacaatgtccagatgcatctgtgtata
tggcttatcgcttgaccagggtaatcgttagctataatctggaataacgtcagcagtggaggggcagcaggcagcatcattcgatcatta
aaataatcaataatgtgccaatggagatgatagatgatcatcacccctaggggcttaacatgctcaatttccattgactttgctaccattga
tgtggacataatcggcatactacgttctaaaattgggattctccgaccccgaaatataagtaaaatgcgtttcgcgagattacaataaagc
gtttcaaaggaagctttcatctatgagatacgtagatacaatagatcttagcaacttataaaggccacgcttgacatcattcatttaccgac
ttaccccattctggacttttctcgtttgcatctttcgcgtggcaatccaggtttttactttggaactacctgatcttgatccccagctggtagaa
agttcgttagcccgtgcagctgcggcaggtactagaattttcctgtaatttcagtttttccactttaattgcggcaattaaagttggtcggttg
cattttgatctatgtatgtatgtagggccatttgattactcagcacgcagcgtgtctaatttggactttaaaatccgttgtgcactttcgggtc
ccaagtacaggcaggtaggtatacaaaaagtacagaagcgtttcacagacaaaagacgttctgttccgccgacagattagatttgtgaa
agttcgtactcgttttcgggtttgccaacagtgcgacacaaaaaggtgcaactggtggtattggttttatattggataaatgcaaactatgt
aaccagggcatttaatttgcaaattgcgttatcattgtgaagcaccccacttcctttctaatcaaaatccacagtgcttagatacttagata
cttcaatactaacttgctaattatattactattgtcgcccgcttcatttgctttgtactcgaaatagctgctccaatccaagagttattcagat
agggtgttggaaaaacacctcatctgactctttattaaacctattcattcatgacgactcttttatggccctatttattgggtgttcaccgcac
ttcctctttgttgttggcactcgaaccacttggacacgagtttgcggatcgccacgcgtacttgtcgccgtatcgtatgacgactctatcact
atatataccataccataaatatacatcgaaatatatttatgaatatggcagatatcttcccatttcaccgccaagtggattgccccttcaga
taatcgcaatttccctcttcgaaatgcaattcatgccttagtgacagagcccttaaaaacaaatcgttatcagttggtggaaattgccgtgt
ttcgatggtttctcccttttttttcaagcgttggtttctaaatgcaaatctgtaaatacgatcattatttgttgtttttttttttaagtctacgttt
attttaagtgtgaaagccaatggccgaccttcagtgtgtacggctatctggaaattttagaaatctcaactgcaattcatggtgctgttcgc
atgcgagcacgatcgtacttatgtatggatgtacatgcatacatatatattcattgcttatcggttcgatttacctttatgggcacgttatcac
gggggagtcatgttttgttgccgatttccgtcgattaaagtccaagttgcgggtgtatcagaagttcgcttacgtagtatatggacttctcaa
tggtcgttcgatttcgttgcagctggtaatgcgaaaatatgagtcaagtgcagtttcgcgatttgagtcgcacatcgagctctttgtttgatt
cgaattgatcgctatctatatggatgggcggcactacatatgtatgcatgtaaat**atgtgatgcattc**gattgacgtatttcctattcctatt
accagatgattagaacacacaaagcacacactatctgcacatttacatgtggggctcgcagctgggtcttcgcccaatagcatgtacata
atgctatcagctttgcgggatccacaatacgtggcgtatctgccagtgatctcctccgctcaatcaatcggccatgcaaatggggcttctca
tcattatcgaacgcattattgttgattggcacacttacaaaactaatccacgatgacagttggtcacgagtcgggttttaaaaacgaattcc
ttgaatagaaatttttactagtcgtataatacatcaagatttcgggtttcatatcaatacattcataattgagaaagttacgaatagaaccc

ttggatcttttcgtattttacttgtaccttaattgcatgtaatcactaaccattcttgaaaagatcatacttatgttctacttcaaatcgaatgg
aacaaaaaattgcagctgccacagttcgtggataaaattccagcccacctttctcccaaaaccaccctgaaaaaaaaaggaaatcgaa
acatccaaatgctttccaaaaatccgttgccaagtgaggagaattcatggcattgcaatgtgtgacctatataaataaatgtatttgtgta
acggctgcataaaaggcactcagaaaacttgcatagtcaaggtgataagtatgtgaacttggcataaaattacgtaaatttaaataatta
aaacatttaaataaatgaaattcgtcaaaaaaaaagagtatttaaatggcatttaaagagtttatatattttatgtatatattaaataatct
aatttcaaatgagttaacaagcataacagcttaaaatttttaacgaacttatgtacagttttaaattacttgggcatactgaaatgggaga
ttttagaaatgcaatggcctgaacaataaacaatagttgtctagctgtaataaaattaaaggcggaaatgtaaaaaaaaaagttgtaag
tcctcaccaaataaaaatagaatccaaaggcagacatattgcattcaaaatatcgaaataaagaagtcttacaagtcacaaaataaac
aaaacagaatagtgtaaacataattactttgcacactgtaaacatatgtagcactacatacatacatagttatgcacatacacatgtacat
atatgtatggtattctggttttcgtgctctttttgaatacaaccacaatatcacgtgatgtttttttattcgtttttgttgggtgccctgtgagaag
tgtagagagcggcgaagagcggaaataacttgacttttgggggtcaggccgcggaaatgggggtgtgtgcgtgtcgcgtttctctctctcc
ctctcgttgagagtttgttgccttttcttttgccgtggaaagcaacaactgccactccccttttccctccccctcctctctttctccagaaact
ggtttatttatccattgaagtcgagcccccaaaagaaatggtatttttggtgcagctgtttgttgtgtgcttgtggtatgtgtgtgcgcatgca
tgtggctttagtgctcgcttcgccgttttcctcgttatgttttgtttacgattccgtcgcgttttggtcttcattttccatttgccgttattcacccg
gcctccaaagtaggcgttagttgcacttgacttgcggttatgatacacataacattagacgcccctactgtacattccacgcactgttgcgg
tcatggcaataaaacccattacttttaactaattaaatgcacacaagttgcataatatcagtttttgaaatagttaaaccgaatttggatta
gttgcgtagtataatttgcaaaatatgtgtagaaggctaaaaattgaaggtttaaatgggtttatctgcaaggtttcagtttggtggcagct
ggacgtttgtaattacattgcagtgcaaagttctctcacttgcattccaaatatataatttgaaaatgatgtgcagttctacccgcgattgta
ctttgcttcgaaggttgataggtgctaaacgagttgatttaacccagagcagccgccacgcccacttttctgattctgtttccgttgataaat
gaagctcatttttacacgcagttgctgtttgcctaagcaaaacaaaaacaaataattaagagggcagcacgtaacgtgatagcgttgttt
acttgcatttccctccctagtttattattgtcgctgacaatcaaaagcggaatggcgaaaacaaatttgatataagaccgagaattgggac
ccccgcgagtttctggggttctttgtagttccaaactccacccagaacaatagtggtgttcaatgtcatagcagctttaaatagtttaactg
ataagcgagcgttatgtgacttgtttgttttaactatgcttgttcccaactatatatacatatataaaatcgatggtgtgacataattccggg
gttttgggatggatcccaatcttagcttacgctattccgcgtttcatcttgtctgatggcctgatttccgttttacgtcaatttgttgaatacaa
ttataatgaattcatgaacataacactatatagtgttcgtaataaccttatcataatcattgttgcaggccgttcggcatgcatctaatcaa
aatatttggaaagttcgatcgtgtggcaccgcggggaacttgatttcttatcaacaaggcacatggcgcacgcgaaacttctatttttatgt
gagagcataggttctgaaatgggtttcacaattacacaaatatacaatatagataacaaccaaatactttatacccgaagttcatcataa
agaatttgccaaatgatggagaacatttgtgtttcttgaccataacatatgtatatgtaaatctatttccgaacttgagaaaccatgtttctg
acttcccaatcaactagaacaggttctcgaatcgtacgataccctattatgaaataacccattttcgttgccccattgttgagctgaagttgt
agcatatggtgggatccataattcaatagtcgcttatcagtgccataaactgtgttgggggtcgagtggcacatagaacaagacaaacac
tttcgagccactcaaggttcgtacgtcatttccccccataaacgagctggtaaatggctaacaacaataacactgccaagagcagcactt
gacccttt**ggatgacacaatg**acgcaaaagccgcgggcatcatcacacgtagatcggtaatc**ggatgaggagata**gcccagtgaaccc
gaaaacatcaatatacatataatataagctcgattagcaagggtatacacttaagcacagtcaacgatcttttccaaacgagattttttac
taatagagttatggattgatgtcatatgacatcatgtgcgtttttaacgattcggagttaatagcagttaaaatttctctaaatgccttgggg
gttcgcagcctttgtaatgcaaagttcaattaaaatgtcaattcttactaaaattccccttttttctattgtgctttccag

>CNMa_promoter
caacaaacagggggttcctactggtgacagataagttgataagaattacttggcccttacaataatccagattgctgaacaagaatattatt
actaacaaataacttaaatattgaagtgttttagcaacatcaagcaatactctatcatgaatttggatacatgcgaggatattacaattcagc
gattacacatgttcaaagaagtgaggaaagcttatttctgaggtactctacaaccacaacatctaacactggcagtgttgcaaagcggttaa
gtccggctttggcggcaattcaaactggttttatgcgaccgtatcaataaagttcaatttagtgcattgtttatggaagtttgagaaagttatcc
gacagatgactcagtgaagcagccacatcggctgtcacgtgatatcctcaactcgagtggctgataattggcggcacgtgctggtgcctttg
ttccggcaagcagtgtcggttcagtcggtcgcgttttaataagtgtcaattagcactgatatggctgccacttaatttgctatcagctctgggc
ggtgtccaaagagcccgcttatcagaatcagtcggacaagtgatgagcgagacacccccgccggatttgggttcggaatagaggtgatga

aaccataaatttcaatttaatcacaaagtctgcggaagaagcgtgggctaccttccgatattttcggatcagacaagaggtttcgagagttc
ggatcatccaattatagactagacggaattaattgctgtctactgggactttgcttttaagacgcattatatttgggaaatgactcgggggag
attctatataagcaaaacacaatctttaaaatacgtcgaaaattttaattaaagatttttatgcgacggcacccccaattaatttgggtttctgt
gtgacttaatccgggcaaggtgttgcgtcatattttttggatttttataatgaagatttgccgagatacgcgaatgattaggtacgcataaga
gataagagatcggtgtagttatctatgcgatggacatgtgtaaatttccatgctattttcgtataaactttattttaaccccttgtatgataga
ttaaacatttttatttagatttgtgaaaggcacatcccacaatgggttggttctatttacaagtgcccaattaatcttaatgtctcattaaaaata
ttttatttacatttgtgacaggttcaccctgcacaatgggtcatatttacatgtacccaaaatgtgtaaggaactcaaatcatgtgatgatttca
tttgaattccatgagtttggaaatgccttttgcgatctcagcttccgctgttgaaaaactgcaagtcgacattgacccaattaacatttaaaat
attccagctaagtcatgacgagtttaatgacctacagttgaagtactggttcatgtgggaacattttccgaatctcaccctggcactatcatcc
cagtgcacagactataaaaaccttgctcaaatcaccttgaagtcccaaagccctgagttaacgaaccgggcgaggg

>CNMa_intron1
gtgagtttgtattactaactattaaatcgaaactagaactgtaaaaaacttactcaaagttataattatcttgtggcacagattatccttggatt
ttcctatactctaaatatttgtacatttaaatgtaatcattgagtttcagctgaatggcgcccattttagcgcccaaatttacgtcgactcattga
taatgaagctttatatataaattgagttatttatacattatcagcagaggttttcagaactgtcactgcgggattttcgattttaattacgttcca
catatagaaaataagtgtagtcgtcttatcagggctgcaagcgaaaggcgaaatcctaaagtttcaatggaaagagtcgcgtgaatcgctg
gaaggtattgcgtagttttacttgggaggggggattccaacttagattcggatgcagatagcttttgtttacacagcccgagaagcgcgga
aaaaaagagggcgtgtcagtagatttgaacctattgttcttgataagatcgagctcctagctctccatgttgcttgtggtccgatattttatgac
gcctccgatgacgcactttatggcgggcaagtaaaagaataagaacgattttattattatatatgcccattcccagttgatgatacgatttgg
gtcatagaaggcgagacccccagcctagtttgatttttaatatgtcagtgaattgcttcgggattctgagcatagattaggttttatttgacgt
gcgaacgaaatgattgtattattggatgatcttctagaatgtaccagtcaaaactggcgatataacaaatatttacatgtgttcattcaagtat
ttggcactctatgattaatatcgtagctatagatgtgcgtacttcttggcagcataattagcaatttagtaactacatttagtcaactatacctt
gacatcttgttatcgcttatcggacaccgttcggtttcagtttgtaataaccgcgaagccgtcatggaaaactgtcaataaattcccatatgt
agttataccagttagcactatatgtttatgggggaagagccacgccccgtgggttcaatggaacctctacgagcgaggccttggctccaac
accaaaatagagatgactcctggctggggatcgcaaattagccacgacaccagtgcattatcagctctagaggcatttttcatattcattgg
gccattaaaatgcgtttggcgtgcgattactctgcgacataaattaccgatcgagatgcgaaaaccgctgacacgatcgccagagtttatcg
cttgcactcgctgtgaaaaaagtgaataaaaatagaagaaaatctggttcagtcagcaacaccaaatagcgaggtggtgtgctttttcgatt
gttttcggtaataaactgcaataaatggaaaatatgttgcaagtcacctggcgccatagaaaatcaaacagcgaattaagtgaaagatggt
gcggttagggcaataatgctactaagaagccagataaggtgcttttgatatacttttataactccaaataaacgccaatttaacgcaatttgt
ccaattgaaaaaaagcatagctgaatatttgcagcagcatacttacaatagcttacaatagaccgcttttaaaggtcattcttaatttgctttc
caacttgttttaacttctattagtttaacttagctgcctcttctgctcataatcgagccacccatctgacgcaccattcattcatttgctaaccacc
ccaaatgactggccatttttaaaagtcagcgaccatcctccccgaaatgaactcaatccttatttgataatgagcccgccgcttacctttagc
attccggtcactcccgctttccgggctgaagcttaattaggcattaatatttcataaatgtcaattacgagggcatagccatcgccttcccaat
cccactgatgacccgtgtccgccgcacatcaagtgatgatactgcaatgtgacctgaatccgtgaactggcatcaaaagaaagccaaaac
ttttaactggaacacggccccgattccattgagcaggccaacttgttggccaactgcaacgtgacttatttaaagtacgagtgggccgaaac
ccctcgaaacggagcattcacgcatttttgcccagcctcagccacaattcgctttgtttttgccaccttgtgttgtttgtaataggaaaccctttg
cgtaagcactcgagggtcctggaaggtcctgaaagtggagtaggggtgcctgtggcatgtgttgcctaattattcgagcctagaattgttag
tatctacatatttagaatttcgtttaaacttgaggactggcattaaagctaagggctatagcaatatctacacctcgagccaccgaagtgac
ccccttaatgttttgattgacttttgattctccgcag

Sequence of enhancer fragments used to generate GFP-based reporters

>bmm1p^{WT}
atagtcgcttatcagtgccataaactgtgttgggggtcgagtggcacatagaacaagacaaacactttcgagccactcaaggttcgtacgtc atttccccccataaacgagctggtaaatggctaacaacaataacactgccaagagcagcacttgaccctttt**ggatgacacaatg**acgcaa aagccgcgggcatcatcacacgtagatcggtaatc**ggatgaggagata**gcccagtgaacccgaaaacatcaatatacatataatataag ctcgattagcaagggtatacacttaagcacagtcaacgatcttttccaaacgagattttttactaatagagttatggattgatgtcatatgaca

> bmm1p$^{\Delta Atf4}$
atagtcgcttatcagtgccataaactgtgttgggggtcgagtggcacatagaacaagacaaacactttcgagccactcaaggttcgtacgtc atttccccccataaacgagctggtaaatggctaacaacaataacactgccaagagcagcacttgaccctttgccgcgggcatcatcacacg tagatcggtaatcgcccagtgaacccgaaaacatcaatatacatataatataagctcgattagcaagggtatacacttaagcacagtcaac gatcttttccaaacgagattttttactaatagagttatggattgatgtcatatgaca

>CNMa1p^{WT}
caacaaacagggggttcctactggtgacagataagttgataagaattacttggcccttacaataatccagattgctgaacaagaatattatt actaacaaataacttaaatattgaagtgttttagcaacatcaagcaatactctatcatgaatttggatacatgcgaggatattacaattcagc gattacacatgttcaaagaagtgaggaaagcttatttctgaggtactctacaaccacaacatctaacactggcagtgttgcaaagcggttaa gtccggctttggcggcaattcaaactggttttatgcgaccgtatcaataaagttcaatttagtgcattgtttatggaagtttgagaaagttatcc gacagatgactcagtgaagcagccacatcggctgtcacgtgatatcctcaactcgagtggctgataattggcggcacgtgctggtgcctttg ttccggcaagcagtgtcggttcagtcggtcgcgttttaataagtgtcaattagcactgatatggctgccacttaatttgctatcagctctgggc ggtgtccaaagagcccgcttatcagaatcagtcggacaagtgatgagcgagacacccccgccggatttgggttcggaatagaggtgatga aaccataaatttcaatttaatcacaaagtctgcggaagaagcgtgggctaccttccgatattttcggatcagacaagaggtttcgagagttc ggatcatccaattatagactagacggaattaattgctgtctactgggactttgcttttaagacgcattatatttgggaaatgactcgggggag attctatataagcaaaacacaatctttaaaatacgtcgaaaattttaattaaagatttttatgcgacggcacccccaattaatttgggtttctgt gtgacttaatccgggcaaggtgttgcgtcatattttttggatttttataatgaagatttgccgagatacgcgaatgattaggtacgcataaga gataagagatcggtgtagttatctatgcgatggacatgtgtaaatttccatgctattttcgtataaactttatttttaaccccccttgtatgataga ttaaacatttttatttagatttgtgaaaggcacatcccacaatgggttggttctatttacaagtgcccaattaatcttaatgtctcattaaaaata ttttatttacatttgtgacaggttcaccctgcacaatgggtcatatttacatgtacccaaaatgtgtaaggaactcaaatcatgtgatgatttca tttgaattccatgagtttggaaatgccttttgcgatctcagcttccgctgttgaaaaactgcaagtcgacattgacccaattaacatttaaaat attccagctaagtcatgacgagtttaatgacctacagttgaagtactggttcatgtgggaacattttccgaatctcaccctggcactatcatcc cagtgcacagactataaaaaccttgctcaaatcaccttgaagtcccaaagccctgagttaacgaaccgggcgaggg

>CNMa1p$^{\Delta Atf4}$
caacaaacagggggttcctactggtgacagataagttgataagaattacttggcccttacaataatccagattgctgaacaagaatattatt actaacaaataacttaaatattgaagtgttttagcactctatcatgaatttggatacatgcgaggatattacaattcagcgattacacatgttc aaagaagtgaggaaagcttatttctgaggtactctacaaccacaacatctaacactggcagtgttgcaaagcggttaagtccggctttggc ggcaattcaaactggttttatgcgaccgtatcaataaagttcaatttagtgcattgtttatggaagtttgagaaagttatccgacagatgactc agtgaagcagccacatcggctgtcacgtgatatcctcaactcgagtggctgataattggcggcacgtgctggtgcctttgttccggcaagca gtgtcggttcagtcggtcgcgttttaataagtgtcaattagcactgatatggctgccacttaatttgctatcagctctgggcggtgtccaaaga gcccgcttatcagaatcagtcggacaagtgatgagcgagacacccccgccggatttgggttcggaatagataaatttcaatttaatcacaa agtctgcggaagaagcgtgggctaccttccgatattttcggatcagacaagaggtttcgagagttcggatcatccaattatagactagacgg aattaattgctgtctactgggactttgcttttaagacgcattatatttgggaaatgactcgggggagattctatataagcaaaacacaatcttt aaaatacgtcgaaaattttaattaaagatttttatgcgacggcacccccaattaatttgggtttctgtgtgacttaatccgggcaaggtttttgg

attttataatgaagatttgccgagatacgcgaatgattaggtacgcataagagataagagatcggtgtagttatctatgcgatggacatgtg
taaatttccatgctattttcgtataaactttatttttaaccccccttgtatgatagattaaacatttttatttagatttgtgaaaggcacatcccaca
atgggttggttctatttacaagtgcccaattaatcttaatgtctcattaaaaatattttatttacatttgtgacaggttcaccctgcacaatgggt
catatttacatgtacccaaaatgtgtaaggaactcaaatcatgtgatgatttcatttgaattccatgagtttggaaatgccttttgcgatctcag
cttccgctgttgaaaaactgcaagtcgacattgacccaattaacatttaaaatattccagctaagtcatgacgagtttaatgacctacagttg
aagtactggttcatgtgggaacattttccgaatctcaccctggcactatcatcccagtgcacagactataaaaaccttgctcaaatcaccttg
aagtcccaaagccctgagttaacgaaccgggcgaggg

>CNMa2p^{WT}
gtgagtttgtattactaactattaaatcgaaactagaactgtaaaaaacttactcaaagttataattatcttgtggcacagattatccttggatt
ttcctatactctaaatatttgtacatttaaatgtaatcattgagtttcagctgaatggcgcccattttagcgcccaaatttacgtcgactcattga
taatgaagctttatatataaattgagttatttatacattatcagcagaggttttcagaactgtcactgcgggattttcgattttaattacgttcca
catatagaaaataagtgtagtcgtcttatcagggctgcaagcgaaaggcgaaatcctaaagtttcaatggaaagagtcgcgtgaatcgctg
gaaggtattgcgtagttttacttgggagggggggattccaacttagattcggatgcagatagcttttgtttacacagcccgagaagcgcgga
aaaaaagagggcgtgtcagtagatttgaacctattgttcttgataagatcgagctcctagctctccatgttgcttgtggtccgatattttatgac
gcctccgatgacgcactttatggcgggcaagtaaaagaataagaacgattttattattatatatgcccattcccagttgatgatacgatttgg
gtcatagaaggcgagacccccagcctagtttgatttttaatatgtcagtgaattgcttcgggattctgagcatagattaggttttatttgacgt
gcgaacgaaatgattgtattattggatgatcttctagaatgtaccagtcaaaactggcgatataacaaatatttacatgtgttcattcaagtat
ttggcactctatgattaatatcgtagctatagatgtgcgtacttcttggcagcataattagcaatttagtaactacatttagtcaactatacctt
gacatcttgttatcgcttatcggacaccgttcggtttcagtttgtaataaccgcgaagccgtcatggaaaactgtcaaataaattcccatatgt
agttataccagttagcactatatgtttatgggggaagagccacgccccgtgggttcaatggaacctctacgagcgaggccttggctccaac
accaaaatagagatgactcctggctggggatcgcaaattagccacgacaccagtgcattatcagctctagaggcatttttcatattcattgg
gccattaaaatgcgtttggcgtgcgattactctgcgacataaattaccgatcgagatgcgaaaaccgctgacacgatcgccagagtttatcg
cttgcactcgctgtgaaaaaagtgaataaaaatagaagaaaatctggttcagtcagcaacaccaaatagcgaggtggtgtgctttttcgatt
gttttcggtaataaactgcaataaatggaaaatatgttgcaagtcacctggcgccatagaaaatcaaacagcgaattaagtgaaagatggt
gcggttagggcaataatgctactaagaagccagataaggtgcttttgatatacttttataactccaaataaacgccaatttaacgcaatttgt
ccaattgaaaaaaagcatagctgaatatttgcagcagcatacttacaatagcttacaatagaccgcttttaaaggtcattcttaatttgctttc
caacttgttttaacttctattagtttaacttagctgcctcttctgctcataatcgagccacccatctgacgcaccattcattcatttgctaaccacc
ccaaatgactggccattttaaaagtcagcgaccatcctccccgaaatgaactcaatccttatttgataatgagcccgccgcttacctttagc
attccggtcactcccgctttccgggctgaagcttaattaggcattaatatttcataaatgtcaattacgagggcatagccatcgccttcccaat
cccactgatgacccgtgtccgccgcacatcaagtgatgatactgcaatgtgacctgaatccgtgaactggcatcaaaagaaagccaaaac
ttttaactggaacacggccccgattccattgagcaggccaacttgttggccaactgcaacgtgacttatttaaagtacgagtgggccgaaac
ccctcgaaacggagcattcacgcatttttgcccagcctcagccacaattcgctttgtttttgccaccttgtgttgtttgtaataggaaaccctttg
cgtaagcactcgagggtcctggaaggtcctgaaagtggagtaggggtgcctgtggcatgtgttgcctaattattcgagcctagaattgttag
tatctacatatttagaatttcgtttaaacttgaggactggcattaaagctaagggctatagcaatatctacacctcgagccaccgaagtgac
ccccttaatgttttgattgacttttgattctccgcag

>CNMa2p$^{\Delta Atf4}$
gtgagtttgtattactaactattaaatcgaaactagaactgtaaaaaacttactcaaagttataattatcttgtggcacagattatccttggatt
ttcctatactctaaatatttgtacatttaaatgtaatcattgagtttcagctgaatggcgcccattttagcgcccaaatttacgtcgactcattga
taatgaagctttatatataaattgagttatttatacattatcagcagaggttttcagaactgtcactgcgggattttcgattttaattacgttcca
catatagaaaataagtgtagtcgtcttatcagggctgcaagcgaaaggcgaaatcctaaagtttcaatggaaagagtcgcgtgaatcgctg
gaaggtattgcgtagttttacttgggagggggggattccaacttagattcggatgcagatagcttttgtttacacagcccgagaagcgcgga
aaaaaagagggcgtgtcagtagatttgaacctattgttcttgataagatcgagctcctagctctccatgttgcttgtggtccgatattttatgac

gcctctatggcgggcaagtaaaagaataagaacgattttattattatatatgcccattcccagttgatgatacgatttgggtcatagaaggcg
agacccccagcctagtttgatttttaatatgtcagtgaattgcttcgggattctgagcatagattaggttttatttgacgtgcgaacgaaatga
ttgtattattggatgatcttctagaatgtaccagtcaaaactggcgatataacaaatatttacatgtgttcattcaagtatttggcactctatga
ttaatatcgtagctatagatgtgcgtacttcttggcagcataattagcaatttagtaactacatttagtcaactataccttgacatcttgttatcg
cttatcggacaccgttcggtttcagtttgtaataaccgcgaagccgtcatggaaaactgtcaaataaattcccatatgtagttataccagttag
cactatatgtttatgggggaagagccacgccccgtgggttcaatggaacctctacgagcgaggccttggctccaacaccaaaatagagat
gactcctggctggggatcgcaaattagccacgacaccagtgcattatcagctctagaggcatttttcatattcattgggccattaaaatgcg
tttggcgtgcgattactctgcgacataaattaccgatcgagatgcgaaaaccgctgacacgatcgccagagtttatcgcttgcactcgctgtg
aaaaaagtgaataaaaatagaagaaaatctggttcagtcagcaacaccaaatagcgaggtggtgtgctttttcgattgttttcggtaataa
actgcaataaatggaaacacctggcgccatagaaaatcaaacagcgaattaagtgaaagatggtgcggttagggcaataatgctactaa
gaagccagataaggtgcttttgatatacttttataactccaaataaacgccaatttaacgcaatttgtccaattgaaaaaaagcatagctga
atatttgcagcagcatacttacaatagcttacaatagaccgcttttaaaggtcattcttaatttgctttccaacttgttttaacttctattagttta
acttagctgcctcttctgctcataatcgagccacccttcattcatttgctaaccaccccaaatgactggccatttttaaaagtcagcgaccatc
ctccccgaaatgaactcaatccttatttgataatgagcccgccgcttacctttagcattccggtcactcccgctttccgggctgaagcttaatta
ggcattaatatttcataaatgtcaattacgagggcatagccatcgccttcccaatcccactgatgacccgtgtccgccgcacatcaagtgatg
atactgcaatgtgacctgaatccgtgaactggcatcaaaagaaagccaaaacttttaactggaacacggccccgattccattgagcaggc
caacttgttggccaactgcaacgtgacttatttaaagtacgagtgggccgaaacccctcgaaacggagcattcacgcatttttgcccagcct
cagccacaattcgctttgtttttgccaccttgtgttgtttgtaataggaaacccctttgcgtaagcactcgagggtcctggaaggtcctgaaagt
ggagtaggggtgcctgtggcatgtgttgcctaattattcgagcctagaattgttagtatctacatattttagaatttcgtttaaacttgaggact
ggcattaaagctaagggctatagcaatatctacacctcgagccaccgaagtgacccccttaatgttttgattgacttttgattctccgcag
